# Synthesis and reactivity of the di(9-anthryl)methyl radical

**DOI:** 10.3762/bjoc.20.193

**Published:** 2024-09-05

**Authors:** Tomohiko Nishiuchi, Kazuma Takahashi, Yuta Makihara, Takashi Kubo

**Affiliations:** 1 Department of Chemistry, Graduate School of Science, Osaka University, 1-1 Machikaneyama, Toyonaka, Osaka 560-0043, Japan; 2 Innovative Catalysis Science Division, Institute for Open and Transdisciplinary Research Initiatives (ISC-OTRI), Osaka University, Osaka, Japan; 3 Spintronics Research Network Division, Institute for Open and Transdisciplinary Research Initiatives (SRN-OTRI), Osaka University, Osaka, Japan

**Keywords:** anthracene, cation, dimerization, radical, reactivity

## Abstract

The di(9-anthryl)methyl (DAntM) radical was synthesized and investigated to elucidate its optical, electrical properties, and reactivity. The generation of the DAntM radical was confirmed by its ESR spectrum, which showed two broad signals. The unpaired electron is primarily localized on the central sp^2^ carbon and slightly delocalized over the two anthryl moieties. Although the DAntM radical undergoes dimerization in solution, the radical still remains even at 190 K due to the bulky nature of the two anthryl groups. Interestingly, upon exposure to air, the purple color of the radical solution quickly fades to orange, resulting in decomposition to give 9-anthryl aldehyde and anthroxyl radical derivatives.

## Introduction

Organic radicals have garnered significant attention in various research fields, including catalysis [1–4], chromophores [5–8], and as agents in dynamic nuclear polarization [9–12]. Recently, highly stable aromatic hydrocarbon radicals, which can persist in air-saturated solutions for several days to months, have been synthesized by employing bulky substituents around the spin-localized carbon center [13–15]. These stable radicals have paved the way to elucidate the nature of radical species, advancing the field of radical chemistry. However, reducing the reactivity of radical species can mean losing one of their most attractive properties. Therefore, it is very important to explore aromatic hydrocarbon radicals that are sufficiently stable for handling, yet reactive under specific conditions.

**Figure 1:**
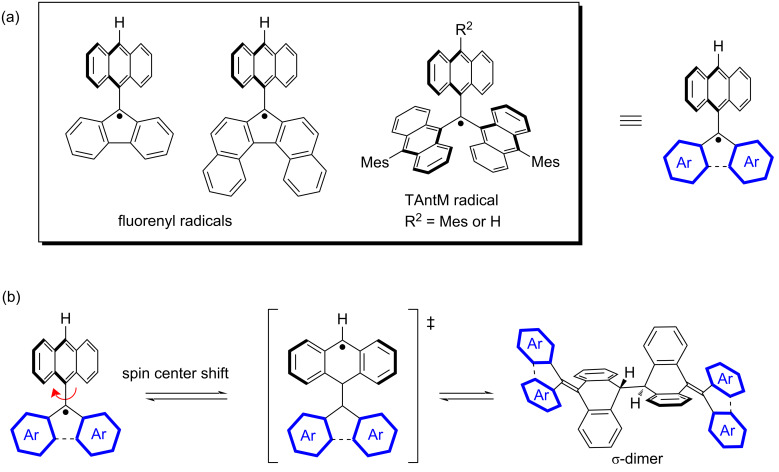
(a) Typical example of stable aromatic hydrocarbon radicals with 9-anthryl units. (b) Tail-to-tail σ-dimer formation by rotating anthryl group and spin center shift.

Previously, we reported aromatic hydrocarbon radicals with 9-anthryl (Ant) units at the spin-center carbon, exhibiting high stability (Figure 1a) [16–21]. Although bulky phenyl substitutions at the spin-center carbon can also provide high stability [13–15], the introduction of an Ant unit allows for spin localization at the 10-position of anthracene through C–C bond rotation, resulting in a tail-to-tail σ-dimer (Figure 1b). The σ-dimer exhibits an equilibrium state between the monomer radical and the σ-dimer in solution, and mechano-stimulus-induced C–C bond fission in the solid state yields the monomer radical [16–18]. Therefore, aromatic hydrocarbon radicals with Ant units possess both stability and reactivity depending on the conditions, giving them high potential for use as reactive catalysts [22–23] and stimuli-responsive sensors [24–25].

**Figure 2:**
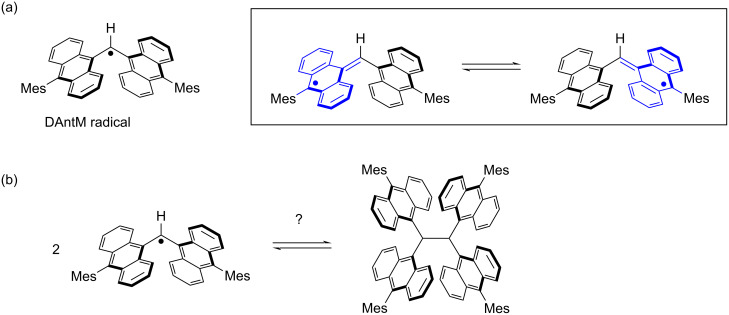
(a) The structure of DAntM radical (left) and its spin delocalization on two anthryl units. (b) Plausible head-to-head σ-dimerization of the DAntM radical.

To further investigate this system, we designed the di(9-anthryl)methyl (DAntM) radical, which lacks one Ant unit compared to the tri(9-anthryl)methyl (TAntM) radical (Figure 2) [17]. By reducing the number of Ant units, we anticipated that the DAntM radical would exhibit spin delocalization between the two Ant units, differing from the basic skeleton of the highly reactive diphenylmethyl radical [26–28]. This spin delocalization is similar to that of the galvinoxyl radical, which shows high stability in air [29]. Thus, the DAntM radical would be a stable radical with a reactive site. Additionally, utilizing the reactive site, head-to-head σ-dimerization of the DAntM radical could yield 1,1,2,2-tetra(9-anthryl)ethane, which is a new anthracene embedded ethane [30] and would be a good candidate for the synthesis of overcrowded ethylene [31–36].

Herein, we report the synthesis and properties of the DAntM radical. The unpaired electron is primarily located at the central sp^2^ carbon, a highly reactive site. The DAntM radical readily reacts with oxygen, leading to 1,2-dioxetane intermediate and decomposition to give anthryl aldehyde and a stable anthroxyl radical.

## Results and Discussion

**Scheme 1:**
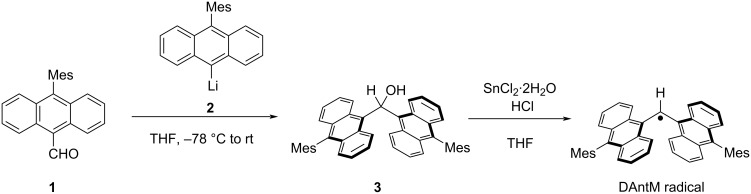
Synthetic route to the DAntM radical.

The synthetic route to the DAntM radical is shown in Scheme 1. The alcohol precursor **3** was prepared via addition reaction of lithium reagent **2** to 10-mesitylanthracene-9-carbaldehyde (**1**) in moderate yield (59%). The generation of the DAntM radical was performed using stannous chloride dihydrate with hydrogen chloride in THF. Upon adding hydrogen chloride to the solution, the solution color changed from orange to deep purple. The presence of the DAntM radical under this reaction conditions was confirmed by ESR measurement.

**Figure 3:**
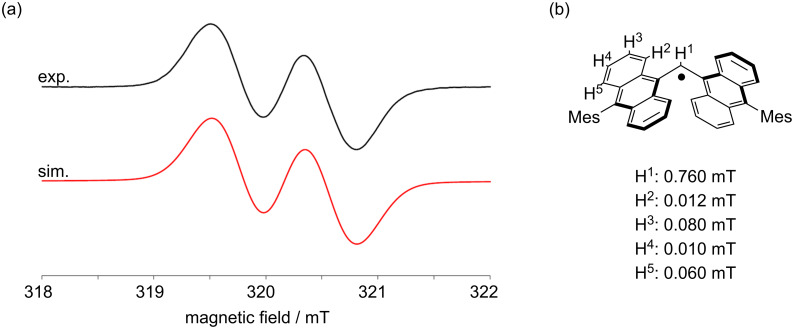
(a) ESR spectrum of the DAntM radical (black line, Exp.) and its simulated pattern (red line, Sim.). (b) Hyperfine coupling constant of the DAntM radical.

For the ESR measurement, a sample was prepared by taking an aliquot from the reaction solvent to ESR tube, evaporating it, and then dissolving it in degassed toluene. The ESR spectrum of the DAntM radical displayed two broad signals with *g* = 2.0028 (Figure 3a). The simulated spectrum indicated that the unpaired electron mainly locates at the central sp^2^ carbon but is slightly delocalized over the two anthryl moieties (Figure 3b, Supporting Information File 1, Figure S1). DFT calculations for structural optimization revealed that the energy difference between two DAntM radical structures with different spin positions, spin localization at the central sp^2^ carbon and on the anthryl group, is small about 1.18 kcal mol^−1^ (Supporting Information File 1, Figure S2). To investigate the activation barrier of this equilibrium, potential energy curve by changing the dihedral angle θ of one anthryl group was calculated. The transition state was calculated with the dihedral angle θ = 30.6° and the activation barrier is only 2.94 kcal mol^−1^ (Supporting Information File 1, Figure S3). Thus, these two structures are likely in equilibrium and rapidly exchange with each other in solution. The energy difference between DAntM dimer (head-to-head σ-dimer) and DAntM radical monomer was also evaluated, showing that the dimer form is energetically preferable by about 3.97 kcal mol^−1^ (Supporting Information File 1, Figure S2). In VT-ESR measurements at low temperatures, the ESR signal integral decreased with cooling (Supporting Information File 1, Figure S4). However, even at 190 K, the relative signal integral compared to that at 295 K remained 0.56. Thus, the σ-dimer formation occurs but the σ-dimer readily dissociates, probably due to the steric bulkiness of the two Ant units [37].

**Figure 4:**
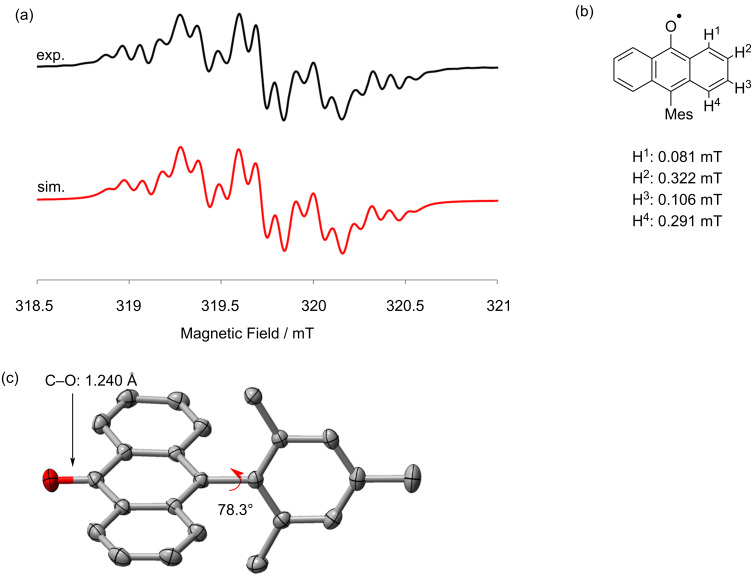
(a) ESR spectrum of anthroxyl radical **5** (black line, Exp.) and its simulated pattern (red line, Sim.). (b) Hyperfine coupling constant of **5**. (c) X-ray structure of **5**. Hydrogen atoms are omitted for clarity.

**Scheme 2:**
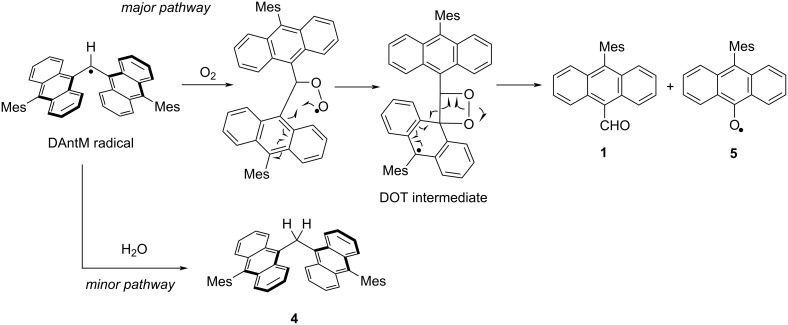
Decomposition pathway of the DAntM radical under air conditions.

It is noteworthy that the purple colored solution of the DAntM radical immediately fade to orange when exposed to air, indicating that the high reactivity of the central sp^2^ carbon. To evaluate the decomposition pathway, the decomposed materials were characterized. Surprisingly, the major compound detected by ^1^H NMR measurement of the crude material was compound **1**, along with di(10-mesityl-9-anthryl)methane (**4**) as a minor product. After silica gel column purification, the isolated yield of these compounds were 64% and 13%, respectively. Additionally, a radical species, showing an ESR peak pattern distinct from that of the DAntM radical and mainly splitting into five peaks with *g* = 2.0037, was confirmed (Figure 4, Figure S7, Supporting Information File 1). ESR and MS measurements as well as X-ray crystallography revealed that the radical species was assigned 10-mesityl-9-anthroyxyl radical (**5**), obtained in 47% yield (Figure 4c, Figure S8, Supporting Information File 1). Thus, two decomposition pathways were considered: a minor pathway involving hydrogen abstraction from water yielding **4**, and a major pathway involving oxygen addition to the central carbon to afford 1,2-dioxetane (DOT) intermediate. Usually, DOT derivatives are known to readily decompose [38], and this DOT intermediate is also considered to decompose upon C–C and O–O bond cleavage to give compounds **1** and **5** (Scheme 2).

**Figure 5:**
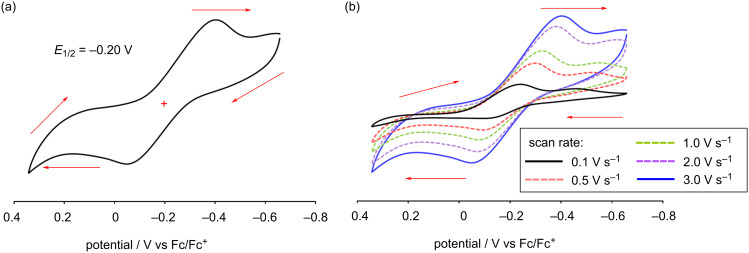
Cyclic voltammogram (CV) of DAntM cation. (a) CV measured with scan rate at 3.0 V s^−1^. (b) Scan rate dependency (0.1, 0.5, 1.0, 2.0, and 3.0 V s^−1^) of the redox wave. Measurement conditions: 100 mM *n*-Bu_4_NPF_6_ and 1 mM DAntM cation in CH_2_Cl_2_. Red arrows indicate the sweep direction.

Owing to the high reactivity of the DAntM radical, cyclic voltammogram (CV) was measured by using the stable DAntM cation, prepared from compound **3** oxidized by antimony(V) chloride, which can be characterized by ^1^H, ^13^C NMR, and UV–vis spectroscopy under ambient conditions. The CV of DAntM species showed a reversible wave at *E*_1/2_ = −0.20 V (V vs Fc/Fc^+^) (Figure 5a) [39]. This redox potential is close to that of TAntM radical and cation [17]. Additionally, at a scan rate of 0.1 V s^−1^, the current peak intensity on the anodic side (from radical to cation) was significantly lower than that on the cathodic side (from cation to radical), resulting in an irreversible redox wave. However, by increasing the scan rate, the current peak intensity on the anodic side gradually increased, and the difference in current intensity between the anodic and cathodic sides became smaller, resulting in a reversible redox wave (Figure 5b). This indicates that the generated DAntM radical rapidly decomposes during the CV measurement, leading to the irreversible redox wave at slow scan rate.

**Figure 6:**
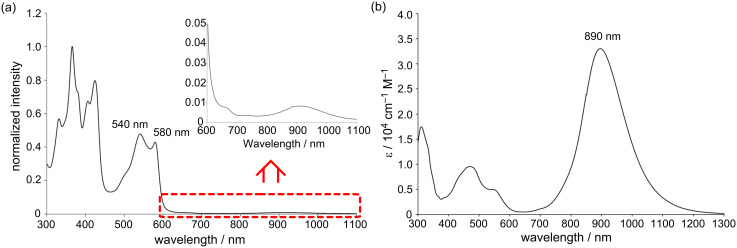
UV–vis–NIR spectra of (a) DAntM radical in toluene, (b) DAntM cation in TFA.

The UV–vis spectra of the DAntM radical and cation were shown in Figure 6a and 6b, respectively. The DAntM radical exhibited a forbidden near-IR (NIR) band centered at 900 nm and relatively intense bands at 580 and 540 nm, whose spectral pattern is similar to the spectrum pattern of the TAntM radical [17]. The result of TD-DFT calculations could reproduce the obtained spectrum shape (Supporting Information File 1, Figure S10). On the other hand, the UV–vis spectrum of the DAntM cation, generated from **3** in TFA solution, showed an intense absorption band at 890 nm, which is the opposite trend compared to the DAntM radical.

## Conclusion

The synthesis and characterization of the DAntM radical were successfully conducted. Although the DAntM radical exhibits σ-dimerization in solution, it readily dissociates into a monomeric radical due to the presence of two bulky 9-anthryl groups. However, the DAntM radical retains a highly reactive nature with oxygen, resulting in the formation of a 1,2-dioxetane (DOT) intermediate and decomposition to aldehyde **1** and anthroxyl radical **5** via C–C and O–O bond cleavage. This reactivity is attributed to the predominant localization of an unpaired electron at the central sp^2^ carbon of the DAntM radical. These findings provide variable insights for the molecular design of readily handled aromatic hydrocarbon radicals that possess both stability and reactivity.

## Supporting Information

File 1 Synthetic procedure and compound characterization data (^1^H, ^13^C NMR, MS, melting point, X-ray crystallography) of new compounds. DFT calculation results and optimized structural Cartesian coordinates.

File 2 Crystallographic information file for compound **5**.

## Data Availability

All data that supports the findings of this study is available in the published article and/or the supporting information to this article.
