# Supplementary material for: Synthesis and reactivity of the di(9-anthryl)methyl radical
Source: Beilstein J Org Chem. 2024 Sep 5;20:2254–60. doi: 10.3762/bjoc.20.193 (PMC11403822; doi:10.3762/bjoc.20.193)
Supplement: File 1 — Synthetic procedure and compound characterization data (1H, 13C NMR, MS, melting point, X-ray crystallography) of new compounds. DFT calculation results and optimized structural Cartesian coordinates. [file Beilstein_J_Org_Chem-20-2254-s001.pdf]

BEILSTEIN JOURNAL OF ORGANIC CHEMISTRY

Supporting Information

for

Synthesis and reactivity of the di(9-anthryl)methyl radical

Tomohiko Nishiuchi, Kazuma Takahashi, Yuta Makihara and Takashi Kubo

Beilstein J. Org. Chem. **2024,** *20,* 2254–2260. doi:10.3762/bjoc.20.193

Synthetic procedure and compound characterization data (^{1}H, ^{13}C NMR, MS, melting point, X-ray crystallography) of new compounds. DFT calculation results and optimized structural Cartesian coordinates

License and Terms: This is a supporting information file under the terms of the Creative Commons Attribution License (https://creativecommons.org/licenses/by/4.0). Please note that the reuse, redistribution and reproduction in particular requires that the author(s) and source are credited and that individual graphics may be subject to special legal provisions.
The license is subject to the *Beilstein Journal of Organic Chemistry* terms and conditions: (https://www.beilstein-journals.org/bjoc/terms)

Contents

1. General information S2
2. Synthesis S2
3. Spin density map of the DAntM radical S4
4. Optimized structures and its relative Gibbs energies of DantM radicals S5
5. Relative Gibbs energies potential curve of the DAntM radical S6
6. VT-ESR study to elucidate the dimerization is solution S7
7. VT-NMR study to elucidate the dimerization in solution S7
8. TLC analysis of decomposition of the DAntM radical S8
9. Spin density map of anthroxyl radical **5** S8
10. X-ray crystallography of anthroxyl radical **5** S9
11. Cyclic voltammogram S10
12. TD-DFT calculation of the DAntM radical S10
13. TD-DFT calculation of the DAntM cation S11
14. Optimized structure of the DAntM cation S12
15. Electrostatic potential surface of the DAntM cation S12
16. NMR spectra S13
17. Cartesian coordinates of optimized structures S17
18. References S22

General Information

All experiments were performed under nitrogen atmosphere. Anhydrous hexane and THF was purchased and used without further purification for reactions. Column chromatography was performed with silica gel [Silica gel 60 (MERCK)]. ^{1}H NMR and ^{13}C NMR spectra were recorded on JEOL ECS-400 spectrometer. APCI–MS spectra were recorded on a Bruker micrOTOF II spectrometer. The UV–vis spectra were recorded on JASCO V-770 spectrophotometer. The ESR spectrum was recorded on JEOL JES-RE1X spectrometer. Cyclic voltammetric measurement was recorded on a BAS Model 612D electrochemical analyzer. The cyclic voltammogram was recorded with a glassy carbon working electrode and a Pt counter electrode in dichloromethane containing 0.1 M n-Bu_4NPF_6 as a supporting electrolyte. The experiment employed an Ag/$AgNO_3$ reference electrode, and was done under argon atmosphere at room temperature.

Computational methods. All DFT calculations were performed with the Gaussian 16 program. For structural optimization, (U)B3LYP-D3/6-31G** level of theory was used. Spin density of DAntM radical and anthroxyl radical were calculated using optimized structure with UBLYP/6-31G** level of theory. TD-DFT calculations were performed using optimized structure with (U)CAM-B3LYP/6-31G** level of theory.

Synthesis

Synthesis of compound 3

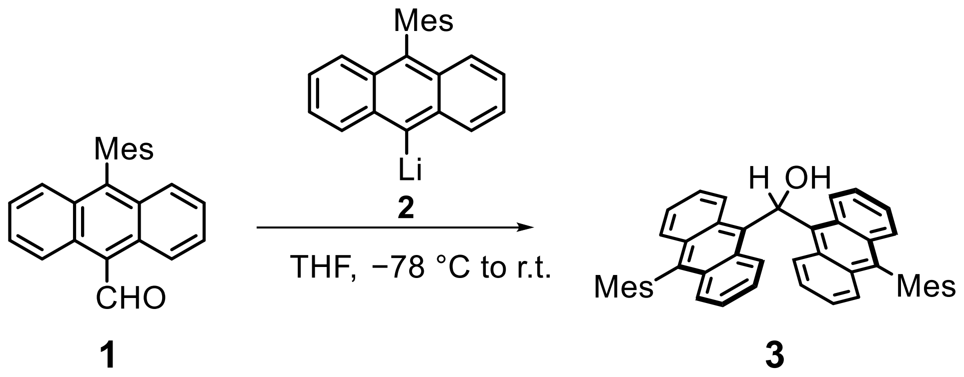

To a solution of 9-bromo-10-mesitylanthracene (751 mg, 2.00 mmol) in THF (10 ml) was added n-BuLi (1.6 M hexane solution, 1.25 ml, 2.00 mmol) at −78 °C. After stirring for 30 min at same temperature, compound **1**[S1](324 mg, 1.00 mmol) was added and stirred for 12 h at room temperature. The reaction was quenched by water, and extracted with dichloromethane and washed with brine. After removal of the solvent *in vacuo*, the crude material was subjected to column chromatography on silica gel (dichloromethane : hexane = 1 : 1) to afford the title compound **3** (365 mg, 0.59 mmol, 59%) as yellow solid.; Mp: 198-200 °C (dec.). ^{1}H NMR (400 MHz, $CDCl_3$) δ 8.71 (d, J = 3.20 Hz, 1H), 8.58 (dd, J = 7.60 Hz, J = 1.60 Hz, 4H), 7.52 (m, 4H), 7.21 (m, 8H), 7.10 (s, 4H), 2.98 (d, J = 3.60 Hz, 1H), 2.54 (s, 6H), 1.73 (s, 12H); ^{13}C NMR (100 MHz, $CDCl_3$) δ 137.83, 137.43, 137.18, 134.95, 134.25, 130.04, 129.93, 128.27, 126.89, 125.71, 125.37, 124.94, 73.16, 21.24, 19.94. HR-MS (APCI) Calcd for $C_{47}H_{40}O$ [M^{+}+H]: m/z 621.3152, Found: 621.3152.

Generation of DAntM radical and elucidation of the decomposition pathway

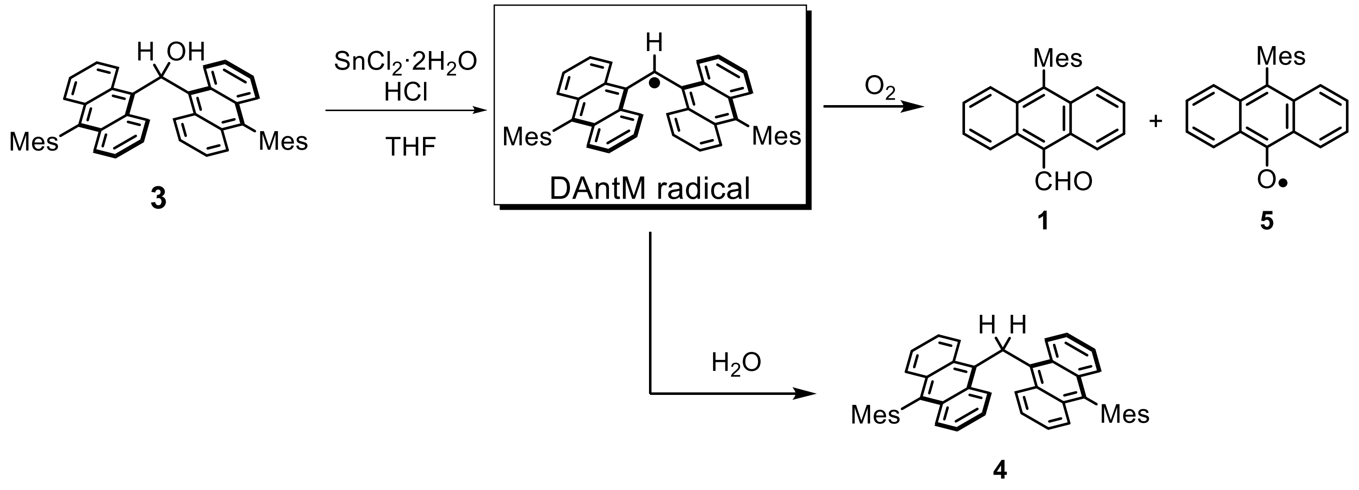

To a solution of compound **3** (200 mg, 0.320 mmol) and stannous chloride dihydrate (109 mg, 0.480 mmol) in THF (10 ml) under nitrogen atmosphere was added 12 M hydrogen chloride (several drops) and stirred for 1 h at room temperature. After exposing air for 30 min, the reaction was quenched by water, and extracted with dichloromethane and washed with brine. After removal of the solvent *in vacuo*, the crude material was subjected to column chromatography on silica gel (dichloromethane : hexane = 1 : 2) to afford compound **1** (66.3 mg, 0.204 mmol, 64%) as yellow solid, and compound **4** (26.1 mg, 0.043 mmol, 13%) as pale yellow solid. Compound **5** was isolated by recrystallization from dichloromethane, hexane, and small amount of pyridine (46.5 mg, 0.150 mmol, 47%).

To measure the ESR spectrum, an aliquot of the solution was picked and it was evaporated in the ESR tube. Degassed toluene was added to the ESR tube and freeze-pump-thaw cycling was conducted for five times. To measure the UV–vis spectrum, aliquot of the solution was picked and diluted into degassed toluene in UV cell.

Compound **1**[S1]: ^{1}H NMR (400 MHz, $CDCl_3$) δ 11.61 (s, 1H), 9.04 (d, *J* = 9.20 Hz, 2H), 7.66 (dd, *J* = 6.80 Hz, *J* = 1.20 Hz, 2H), 7.57 (d, *J* = 8.40 Hz, 2H), 7.40 (dd, *J* = 6.80 Hz, *J* = 1.20 Hz, 2H), 7.10 (s, 2H), 2.46 (s, 3H), 2.54 (s, 3H), 1.69 (s, 6H); ^{13}C NMR (100 MHz, $CDCl_3$) δ 193.38, 145.12, 137.77, 136.89, 134.02, 131.92, 129.44, 128.81, 128.41, 126.94, 125.90, 124.72, 123.80, 21.22, 19.88.

Compound **4**: Mp: 243-245°C (dec.). ^{1}H NMR (400 MHz, $CDCl_3$) δ 8.32 (dd, *J* = 8.00 Hz, *J* = 1.60 Hz, 4H), 7.49 (m, 4H), 7.21 (m, 8H), 7.09 (s, 4H), 6.15 (s, 2H), 2.45 (s, 6H), 1.72 (s, 12H); ^{13}C NMR (100 MHz, $CDCl_3$) δ 137.61, 137.05, 135.45, 135.08, 134.20, 130.40, 129.80, 128.23, 126.92, 125.32, 124.95, 124.92, 29.5, 21.26, 19.92. HR-MS (APCI) Calcd for $C_{47}H_{40}$ [M^{+}+H]: *m*/*z* 605.3203, Found: 605.3202.

Compound **5**: Mp: 216-217°C. MS (APCI): *m*/*z* 312 [M^{+}+H].

Generation of the DAntM cation

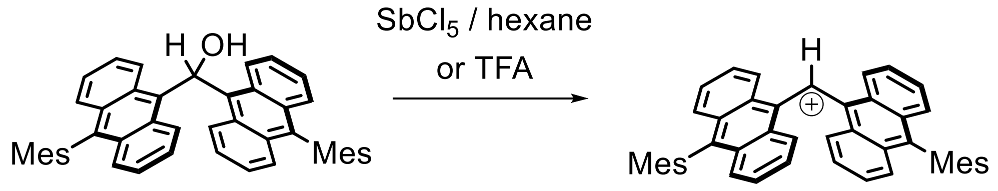

3 DAntM cation

To a suspension of compound **3** (10 mg, 0.025 mmol) in hexane (1 ml) was added a solution of pentachloroantimonate (1.0 M in CH_2Cl_2, 0.1 ml), affording a black solid of DAntM cation. After washed the solid by hexane, residual hexane was evaporated. This solid was used for CV measurement. Due to the highly hygroscopic nature of DAntM cation, melting point measurement was difficult. To measure the UV–vis or NMR spectra of DAntM cation, compound **3** was dissolved in TFA or TFA-*d*, respectively, for *in situ* generation. From the NMR spectra, DAntM cation is quantitatively generated from **3** in TFA-*d*. ^{1}H NMR (400 MHz, TFA-*d*) δ 10.87 (s, 1H), 8.41 (d, *J* = 8.4 Hz, 4H), 7.81 (dd, *J* = 8.4 Hz, *J* = 0.8 Hz, 4H), 7.62 (m, 4H), 7.54 (m, 4H), 7.16 (s, 4H), 2.42 (s, 6H), 1.84 (s, 12H); ^{13}C NMR (100 MHz, TFA-*d*) δ 169.03, 158.04, 142.79, 138.20, 138.04, 137.46, 136.71, 134.59, 134.19, 133.17, 131.36, 130.88, 128.04, 21.90, 20.67.

Spin density map of the DAntM radical

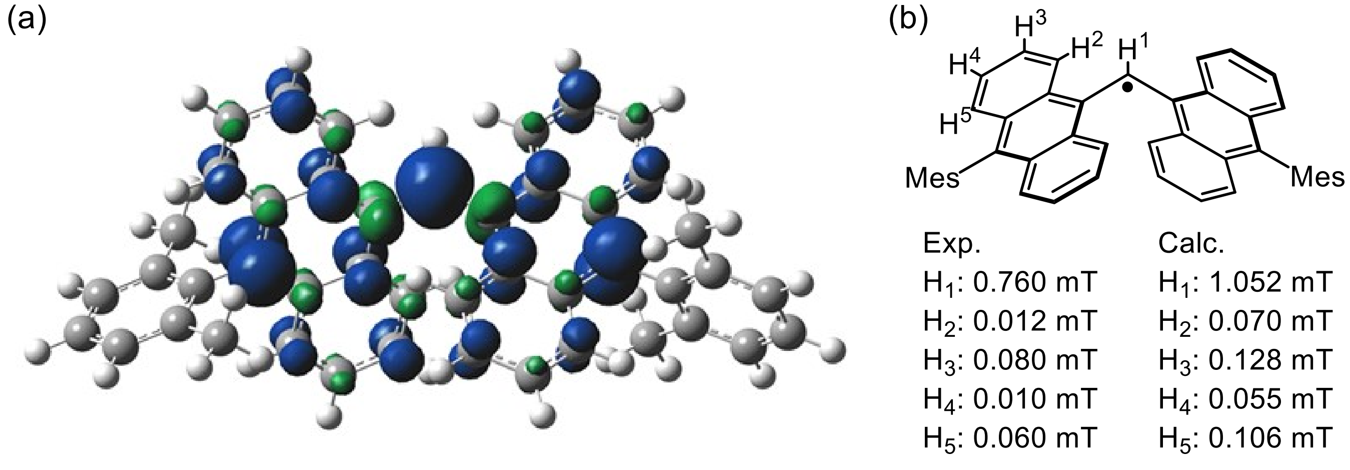

Figure S1. (a) Calculated spin distribution of DAntM radical with spin localization at the central sp^2 carbon. To reduce the computational costs, one methyl substituent of the mesityl group at 4-position was replaced by hydrogen. (b) Experimental and calculated hyperfine coupling constant of **5**. Calculations were conducted by UBLYP/6-31G**//UB3LYP-D3/6-31G** level of theory.

Optimized structures and its relative Gibbs energies of DAntM radicals

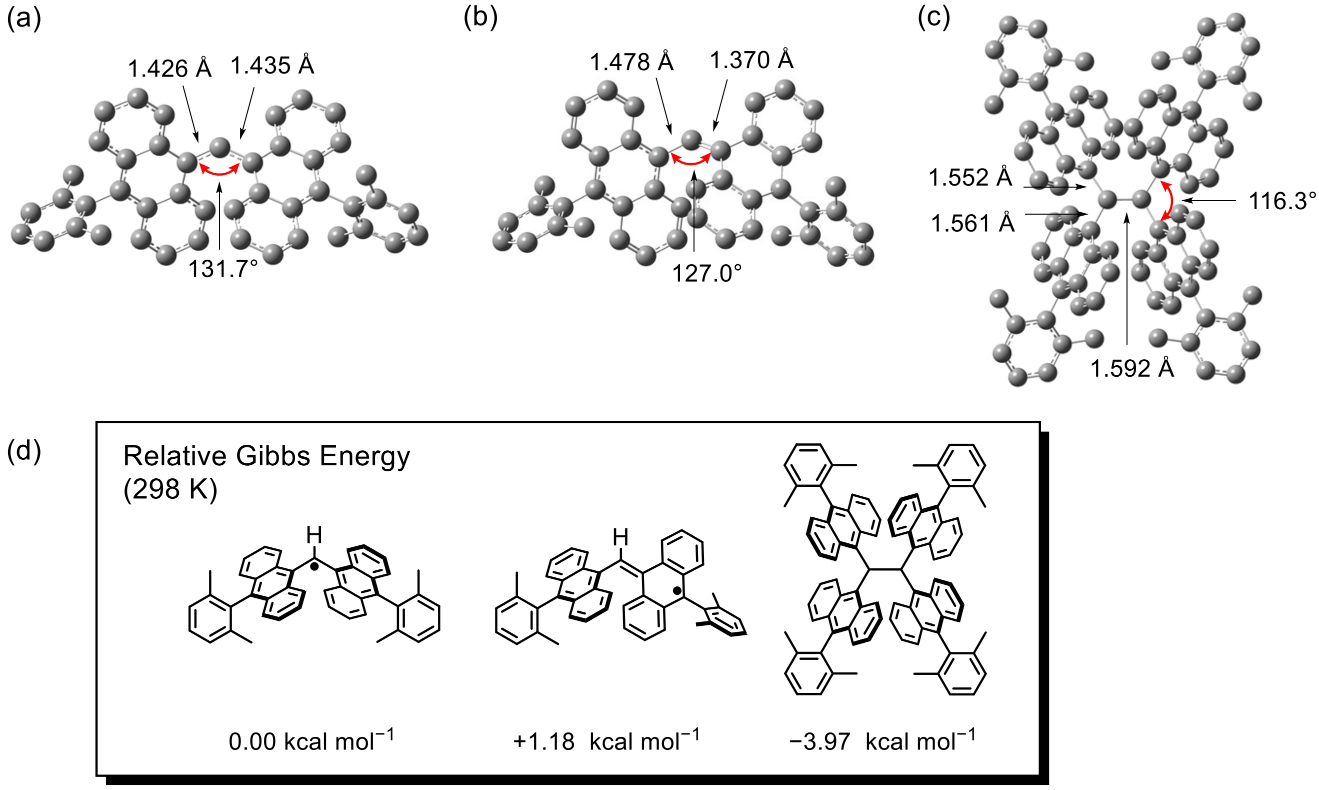

Figure S2. (a) Optimized structure and structural parameters of a DAntM radical with spin localization at the central sp^2 carbon.
(b) Optimized structure and structural parameters of a DAntM radical with spin localization on the right anthryl group. (c) Optimized structure and structural parameters of the DAntM radical dimer. (d) Relative Gibbs energies (298 K) of above structures. To reduce the computational cost, one methyl substituent of the mesityl group at 4-position was replaced by hydrogen. Structural optimization were conducted by UB3LYP-D3/6-31G** level of theory.

Relative Gibbs energies potential curve of the DAntM radical

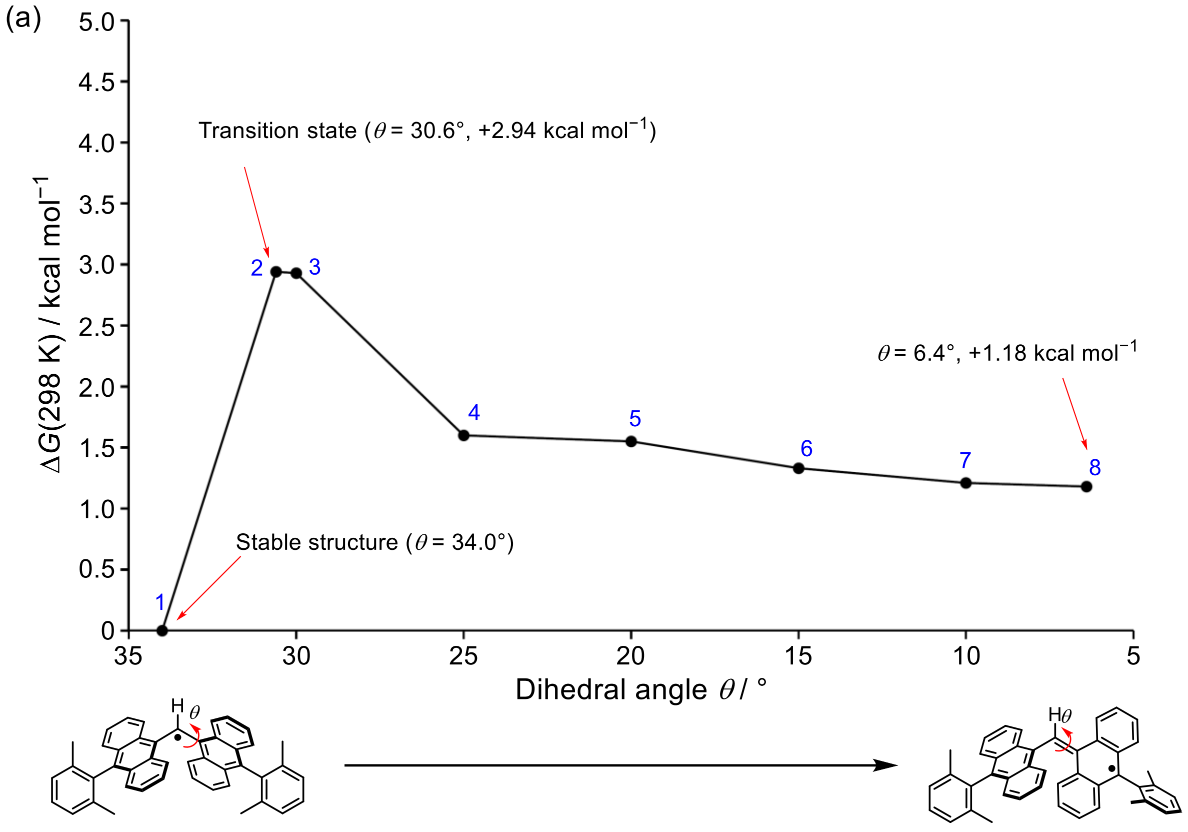

(b)

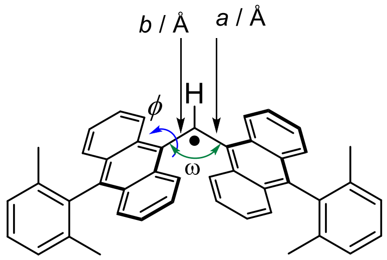

	a / Å	b / Å	ϕ / °	ω / °
1	1.426	1.435	37.9	131.7
2	1.418	1.443	41.3	131.5
3	1.416	1.444	41.9	131.5
4	1.384	1.469	46.8	127.2
5	1.378	1.473	50.7	127.1
6	1.374	1.475	54.4	127.1
7	1.371	1.477	57.9	127.0
8	1.370	1.478	60.4	127.0

Figure S3. (a) Relative Gibbs energies potential curve of the DAntM radical toward dihedral angle of Ant unit. (b) Structural parameters, C-C bond lengths of a and b, dihedral angle ϕ of another anthryl unit, and bond angle ω in each optimized structures from 1 to 8.

VT-ESR study to elucidate the dimerization in solution

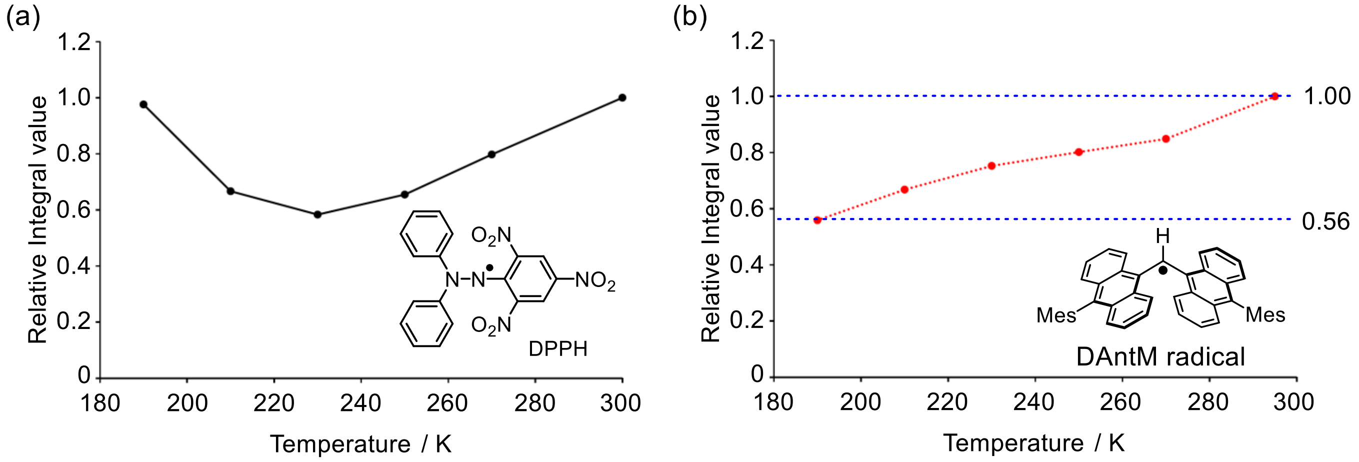

Figure S4. (a) Temperature dependency of the ESR peak integral of 2,2-diphenyl-1-picrylhydrazyl (DPPH) in toluene. (b) Temperature dependency of the ESR peak integral of the DAntM radical corrected by that of DPPH in toluene. Although DPPH is reported not to dimerize in solution, the ESR peak integral shows a temperature dependence due to the Curie law and sensitivity of the instrument;[S2] the relative peak integral decreases to about 0.6 at 230 K and then shows a reverse tendency to increase at lower temperature. Therefore, the dimerization of the DAntM radical was estimated by integral correction using DPPH. Based on these results, we concluded that the DAntM radical shows dimerization in solution.

VT-NMR study to elucidate the dimerization in solution

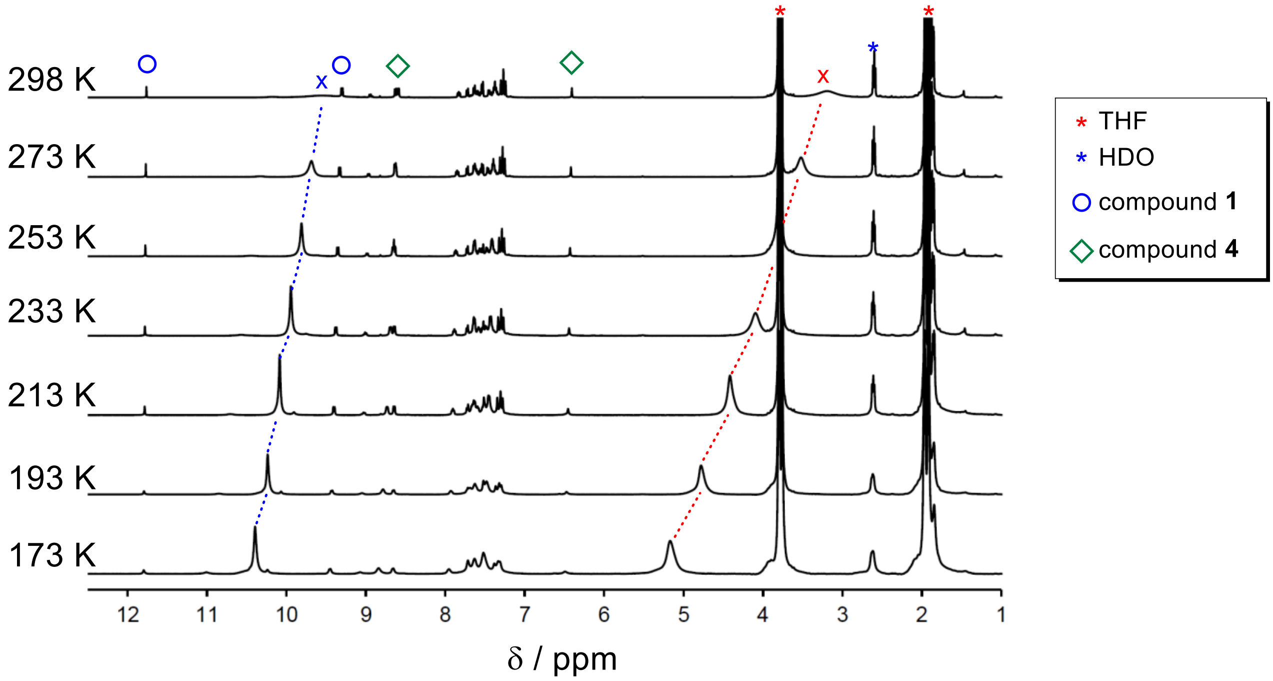

Figure S5. VT-^{1}H NMR of DAntM radical from 298K to 173 K measured by using THF-d_8. For all temperature, decomposed materials of compound **1** (○) and **4** (◇) were observed. Although two broad signals at 9.8 ppm (x) and 3.3 ppm (x) at 298 K became sharpen and shifted to lower magnetic field upon cooling temperature, they are unknown signals and the signals originating from dimer could not be observed probably due to the equilibrium between monomer radical and dimer even at low temperature.

TLC analysis of decomposition of the DAntM radical

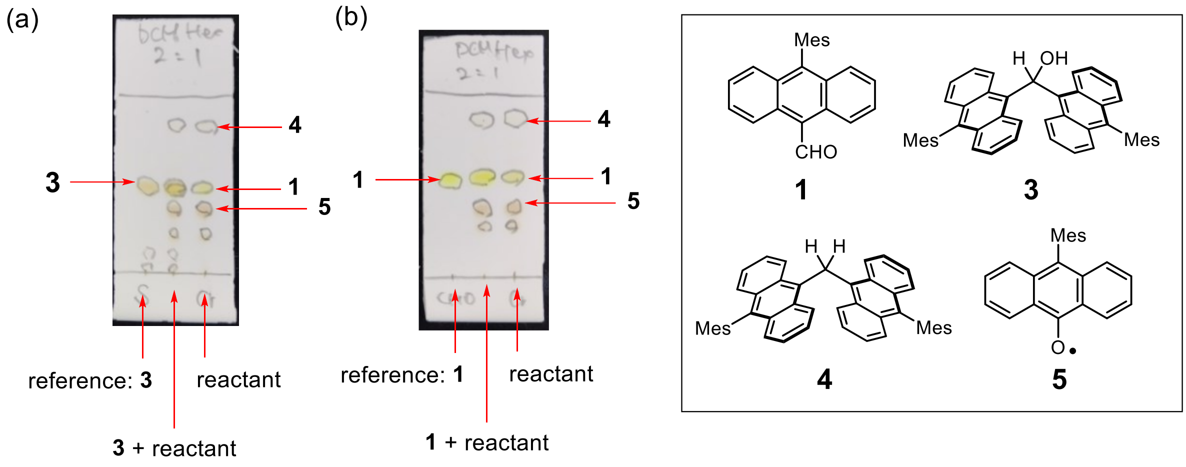

Figure S6. (a) TLC image of reference **3** (left) and reactant for generation of DAntM radical (right), (Developing solvent: dichloromethane : hexane = 2 : 1). In reactant, reference **3** (orange color) was consumed but yellow spot at almost identical R_f value was observed. (b) TLC image of reference **1** (left) and reactant for generation of DAntM radical (right), (Developing solvent: dichloromethane : hexane = 2 : 1). It is obvious that the compound **1** (yellow color) was generated from the reactant.

Spin density map of anthroxyl radical 5

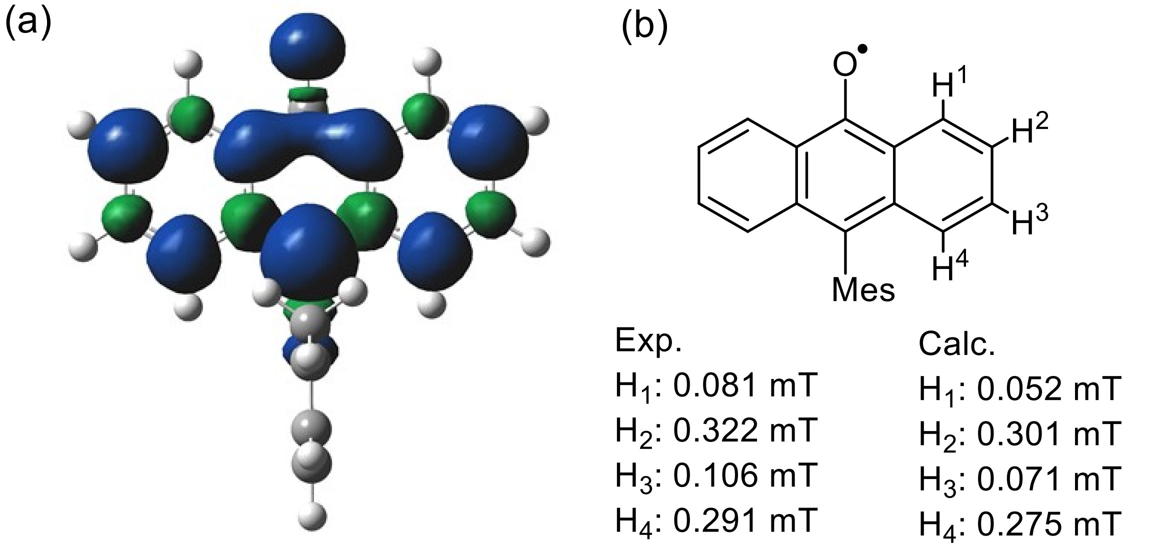

Figure S7. (a) Calculated spin distribution of anthroxyl radical **5**. To reduce the computational costs, one methyl substituent of the mesityl group at 4-position was replaced by hydrogen. (b) Experimental and calculated hyperfine coupling constant of **5**. Calculations were conducted by UBLYP/6-31G**//UB3LYP-D3/6-31G** level of theory.

X-ray crystallography of anthroxyl radical 5

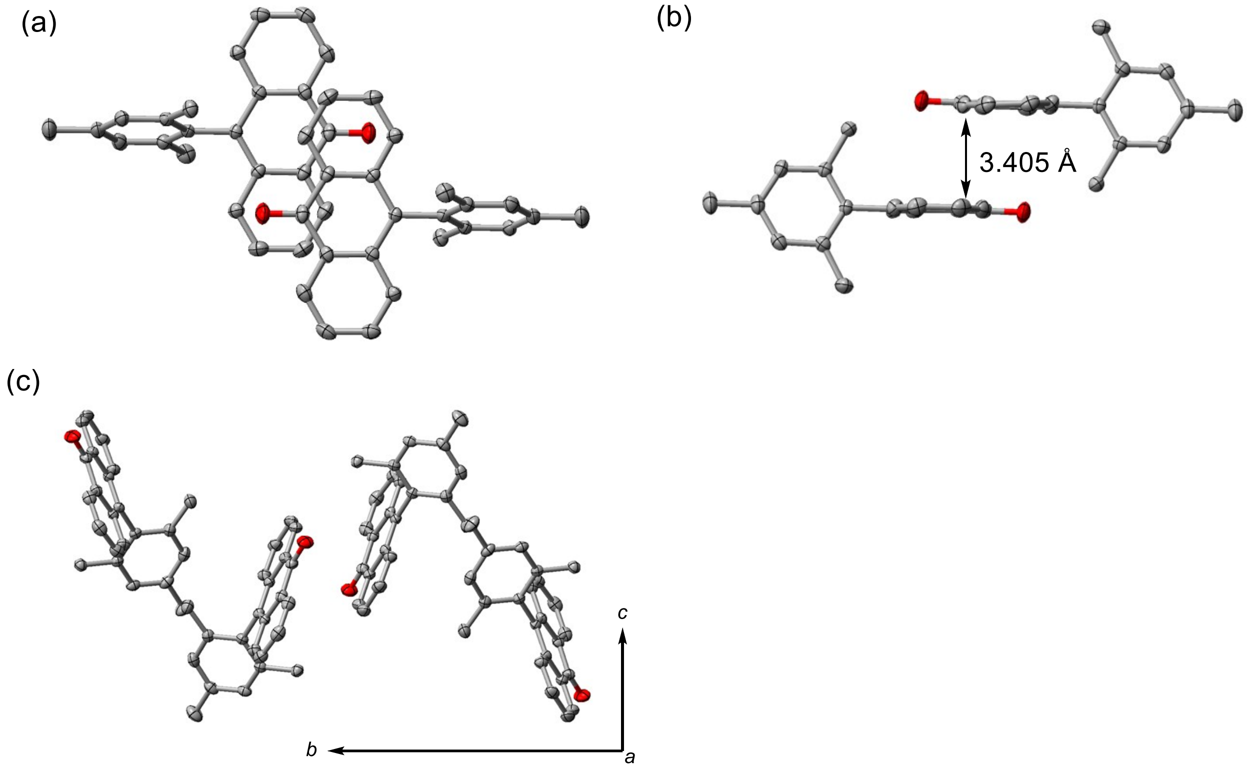

Figure S8. X-ray crystallography of anthroxyl radical **5**. (a) Top view of dimeric structure of **5**. (b) Side view. (c) Packing structure of **5**. Hydrogen atoms are omitted for clarity.

Table S1. Crystal data and structure refinement for **5**.

CCDC number	2373408
Empirical formula	$C_{23}H_{19}O$
Formula weight	311.38
Temperature/K	120.15
Crystal system	monoclinic
Space group	$P2_1/c$
a/Å	8.7514(9)
b/Å	22.1655(16)
c/Å	9.2360(9)
β/°	113.649(12)
Volume/Å^3	1641.1(3)
Z=4	
ρcalcg/cm^3	1.260
μ/mm^{-1}	0.075
F(000)	660.0
Crystal size/mm^3	0.5 × 0.05 × 0.05
Radiation	Mo Kα (λ = 0.71073)
2Θ range for data collection/°	3.674 to 62.224
Index ranges	$-11 \le h \le 11$, $-29 \le k \le 30$, $-12 \le l \le 12$
Reflections collected	19645
Independent reflections	4267 [R_{int} = 0.0333, R_{sigma} = 0.0287]
Data/restraints/parameters	4267/0/220
Goodness-of-fit on F^2	1.061
Final R indexes [I>=2σ (I)]	R_1 = 0.0493, wR_2 = 0.1305
Final R indexes [all data]	R_1 = 0.0637, wR_2 = 0.1378
Largest diff. peak/hole / e Å^{-3}	0.34/−0.23

Cyclic voltammogram

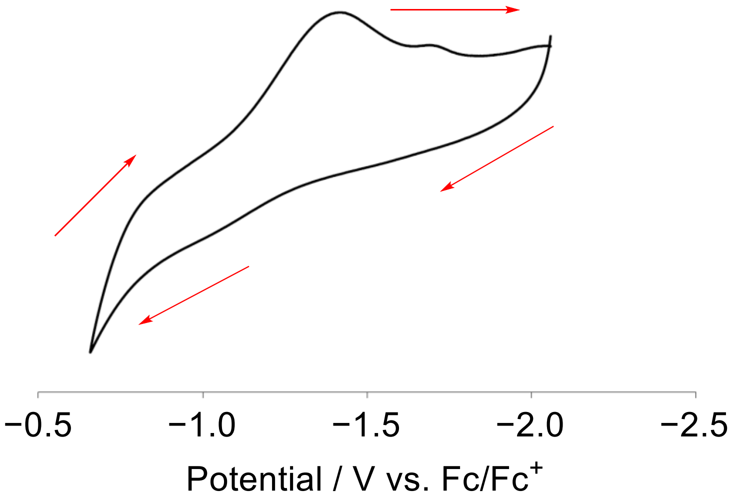

Figure S9. Cyclic voltammogram of the DAntM cation at negative potential field. Measurement conditions: 100 mM n-Bu_4NPF_6 and 1 mM DAntM cation in CH_2Cl_2. Red arrows indicate the sweep direction.

TD-DFT calculation of DAntM radical (UCAM-B3LYP/6-31G**//B3LYP-D3/6-31G**)

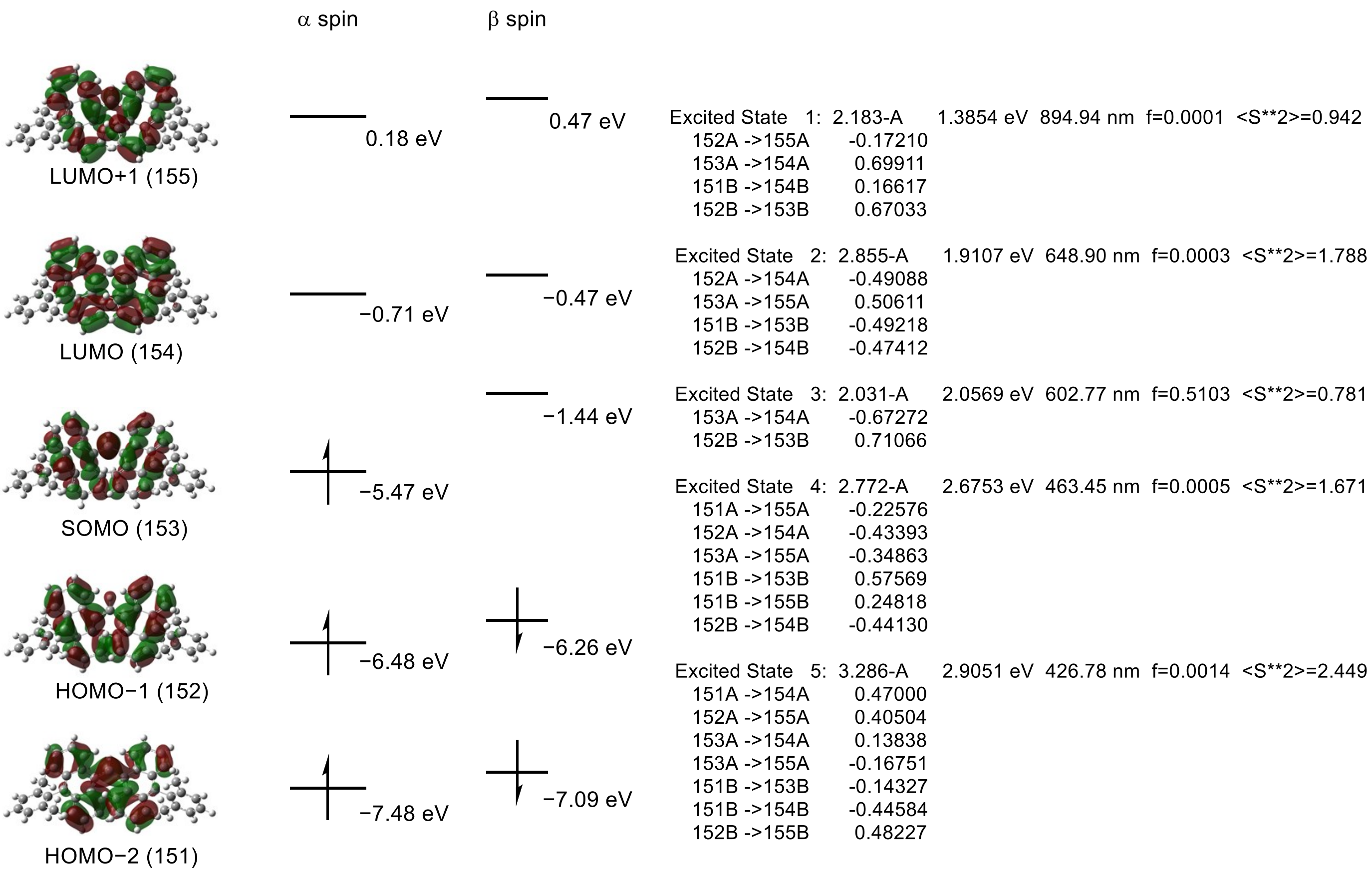

Figure S10. Frontier orbitals, energies, and transitions of the DAntM radical. To reduce the computational costs, one methyl substituent of the mesityl group at 4-position was replaced by hydrogen. Calculations were conducted by UCAM-B3LYP/6-31G**//UB3LYP-D3/6-31G** level of theory.

TD-DFT calculation of the DAntM cation

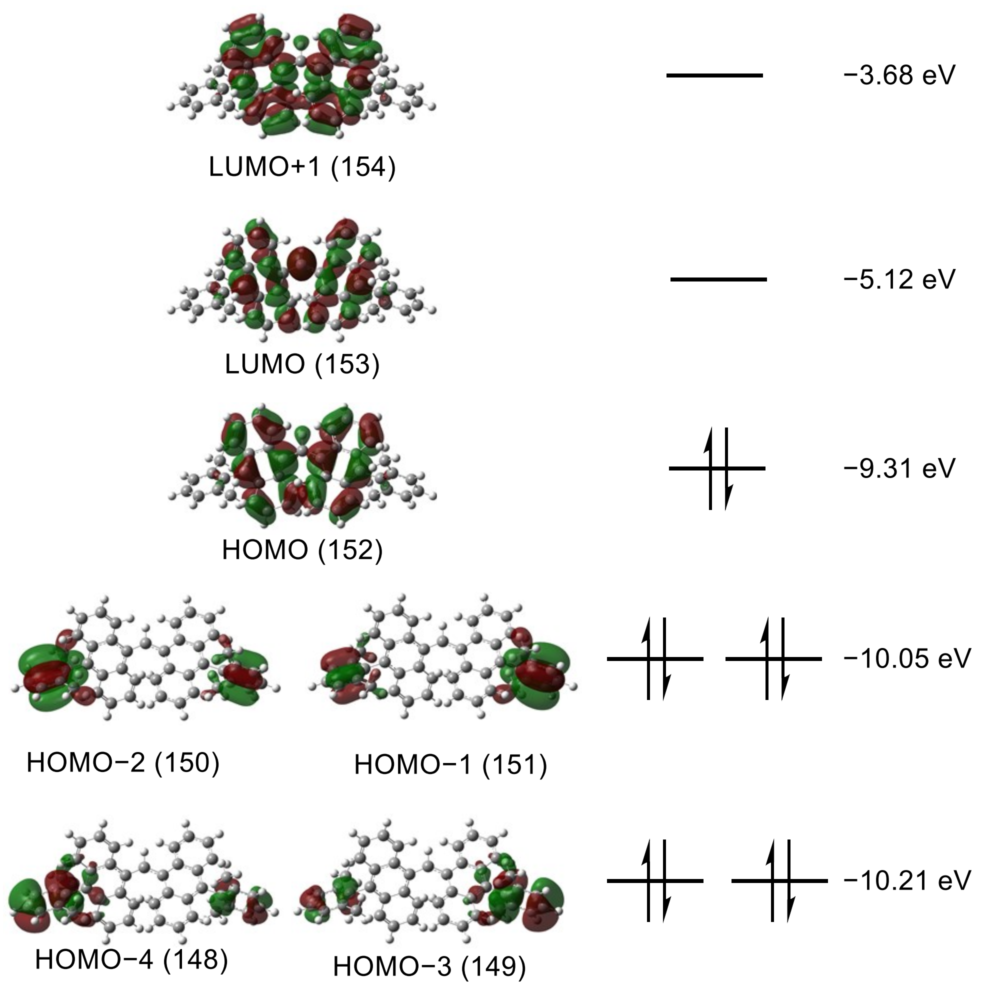


```
Excited State   1:      Singlet-A      1.7245 eV  718.94 nm  f=0.5695  <S**2>=0.000
     152 ->153         0.70237

Excited State   2:      Singlet-A      2.5606 eV  484.20 nm  f=0.0001  <S**2>=0.000
     147 ->153        -0.14797
     148 ->153        -0.24169
     148 ->154         0.19590
     149 ->153         0.54917
     151 ->153        -0.21410

Excited State   3:      Singlet-A      2.5636 eV  483.63 nm  f=0.0001  <S**2>=0.000
     148 ->153         0.54903
     149 ->153         0.24151
     149 ->154         0.19772
     150 ->153        -0.25091
     151 ->153        -0.10253

Excited State   4:      Singlet-A      2.5671 eV  482.97 nm  f=0.0003
<S**2>=0.000
     148 ->153        -0.11583
     149 ->153         0.20343
     150 ->153        -0.28531
     150 ->154         0.19947
     151 ->153         0.55347

Excited State   5:      Singlet-A      2.5673 eV  482.93 nm  f=0.0002
<S**2>=0.000
     148 ->153         0.23606
     149 ->153         0.12646
     150 ->153         0.53980
     151 ->153         0.28123
     151 ->154         0.19509
```

Figure S11. Frontier orbitals, energies, and transitions of the DAntM cation. To reduce the computational costs, one methyl substituent of the mesityl group at 4-position was replaced by hydrogen. Calculations were conducted by CAM-B3LYP/6-31G**//B3LYP-D3/6-31G** level of theory.

Optimized structure of the DAntM cation

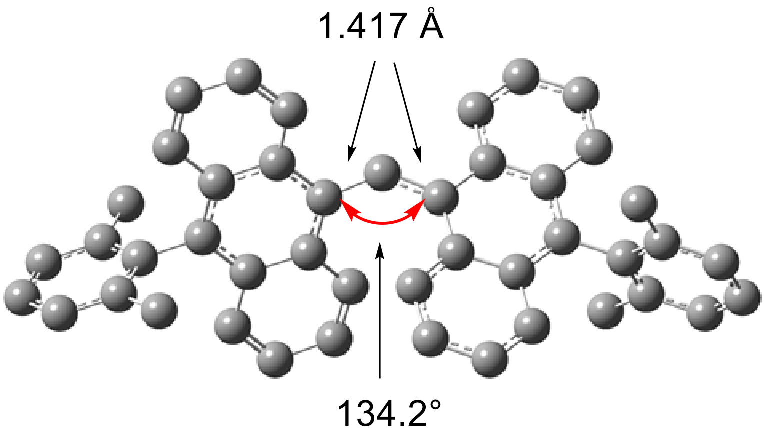

Figure S12. Optimized structure and structural parameters of DAntM cation. To reduce the computational costs, one methyl substituent of the mesityl group at 4-position was replaced by hydrogen. Calculations were conducted by B3LYP-D3/6-31G** level of theory.

Electrostatic potential surface of the DAntM cation

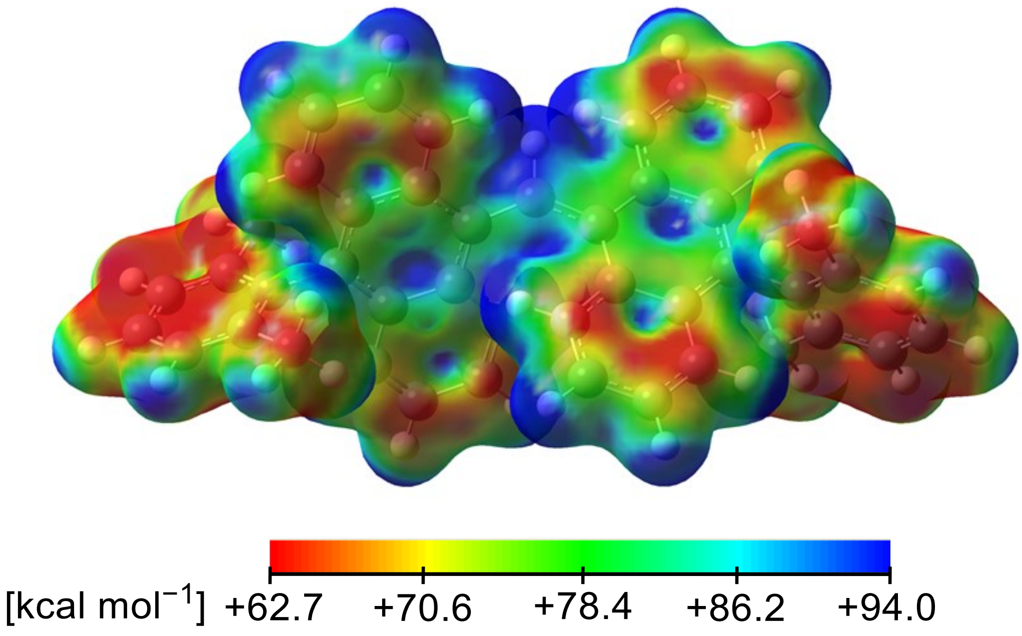

Figure S13. Electrostatic potential surface of the DAntM cation (B3LYP-D3/6-31G**). Energy color range represents from +62.7 (red) to +94.0 (blue) kcal mol^{-1}

NMR spectra

^{1}H NMR (400 MHz) of compound **3** ($CDCl_3$)

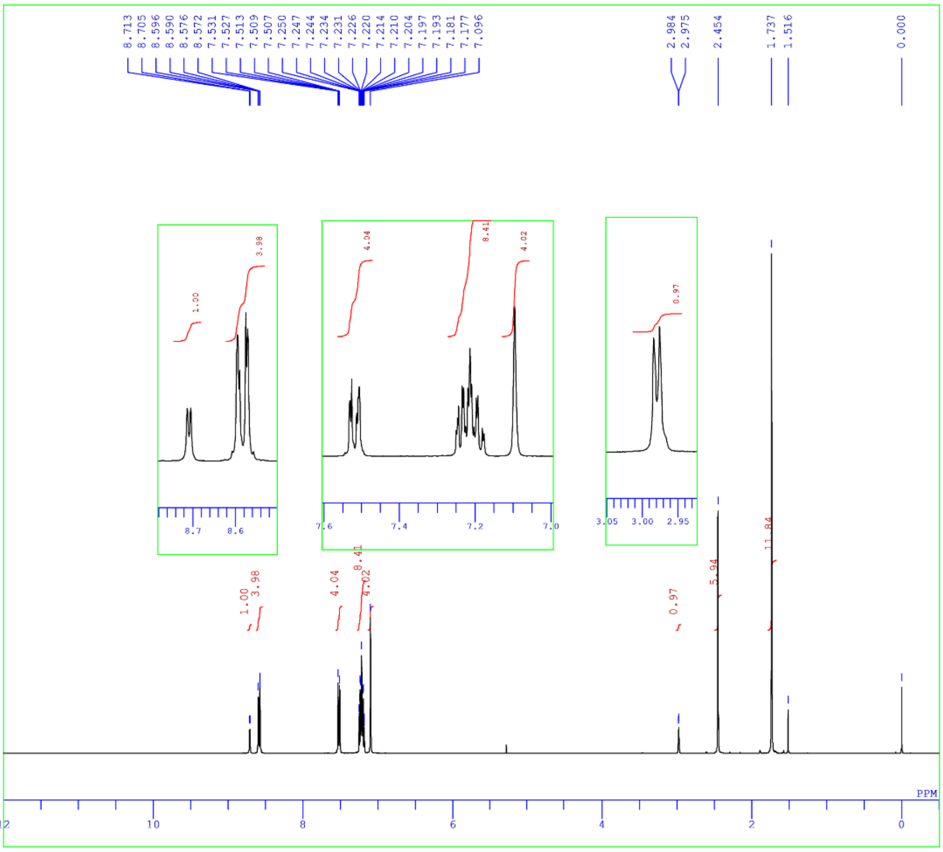

^{13}C NMR (100 MHz) of compound **3** ($CDCl_3$)

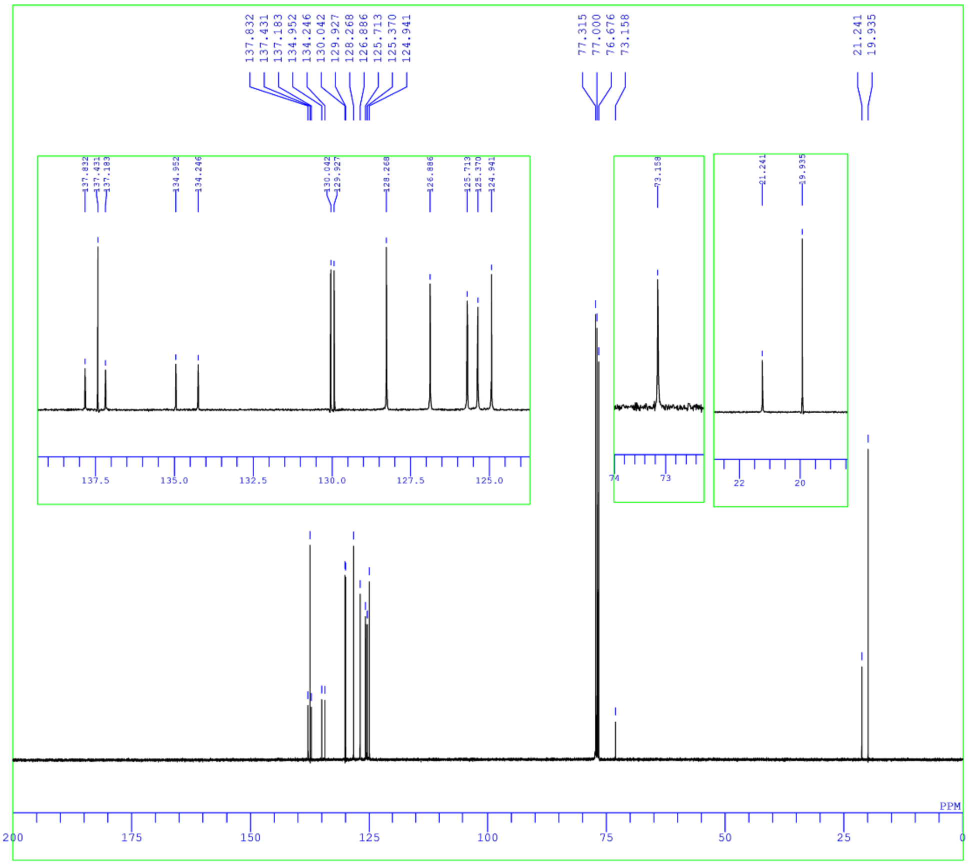

^{1}H NMR (400 MHz) of compound **1** ($CDCl_3$)

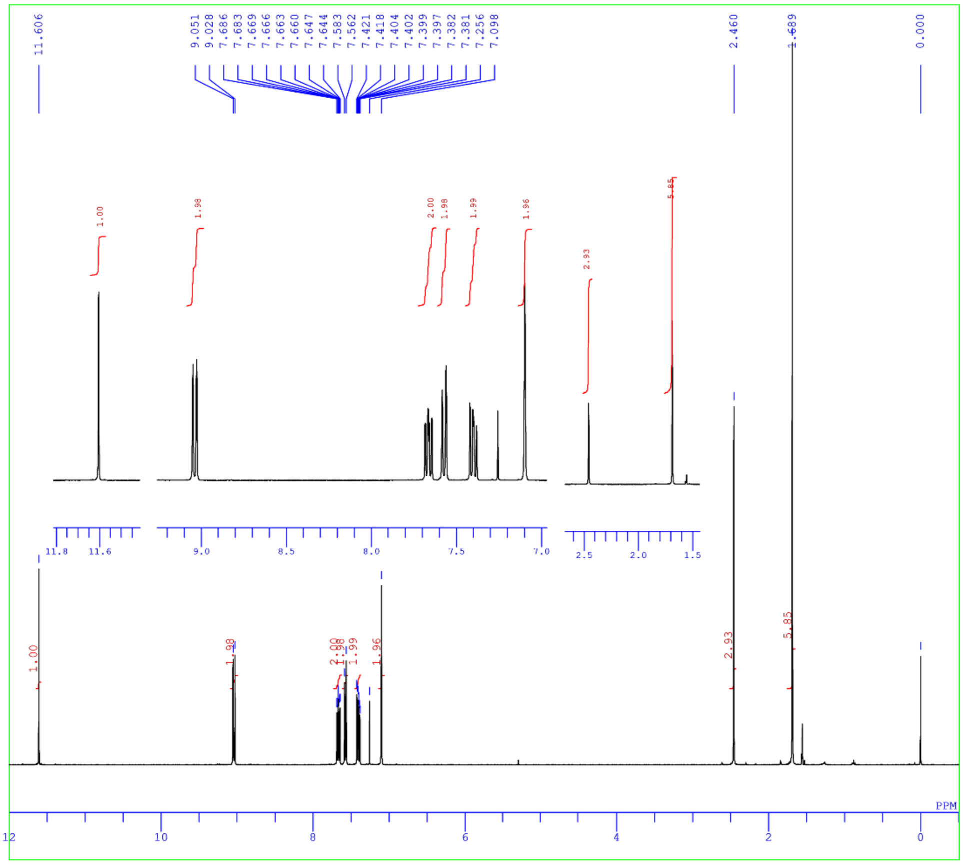

^{13}C NMR (100 MHz) of compound **1** ($CDCl_3$)

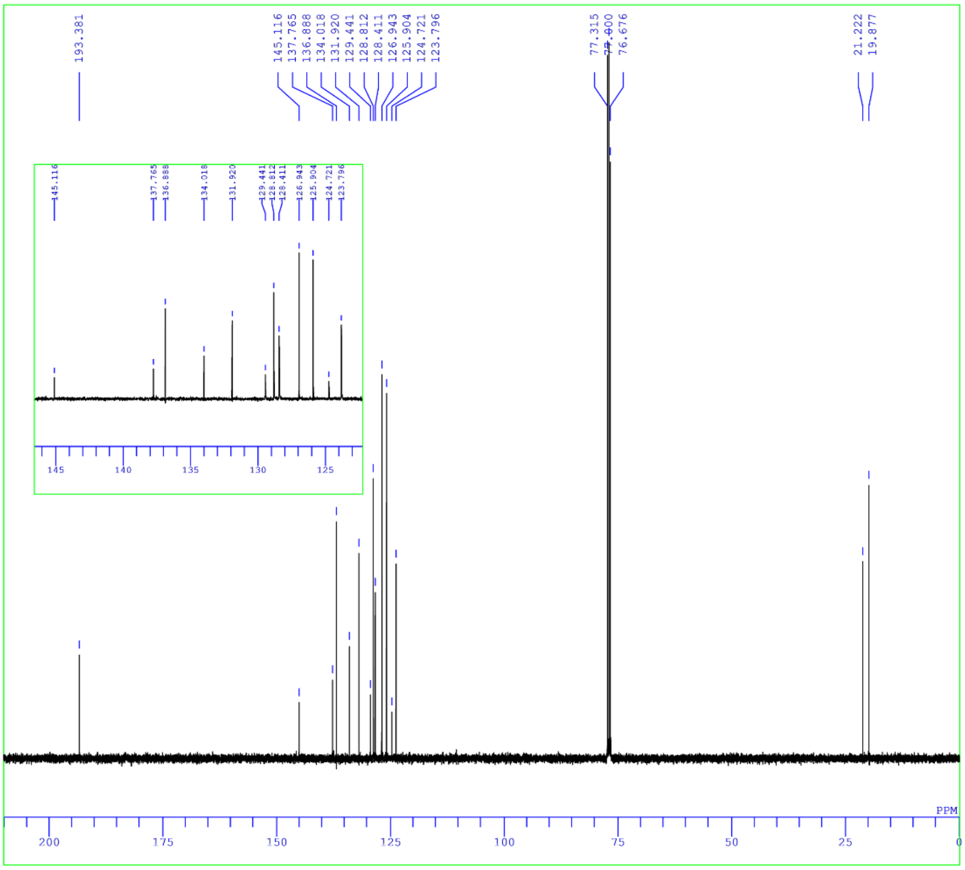

^{1}H NMR (400 MHz) of compound **4** ($CDCl_3$)

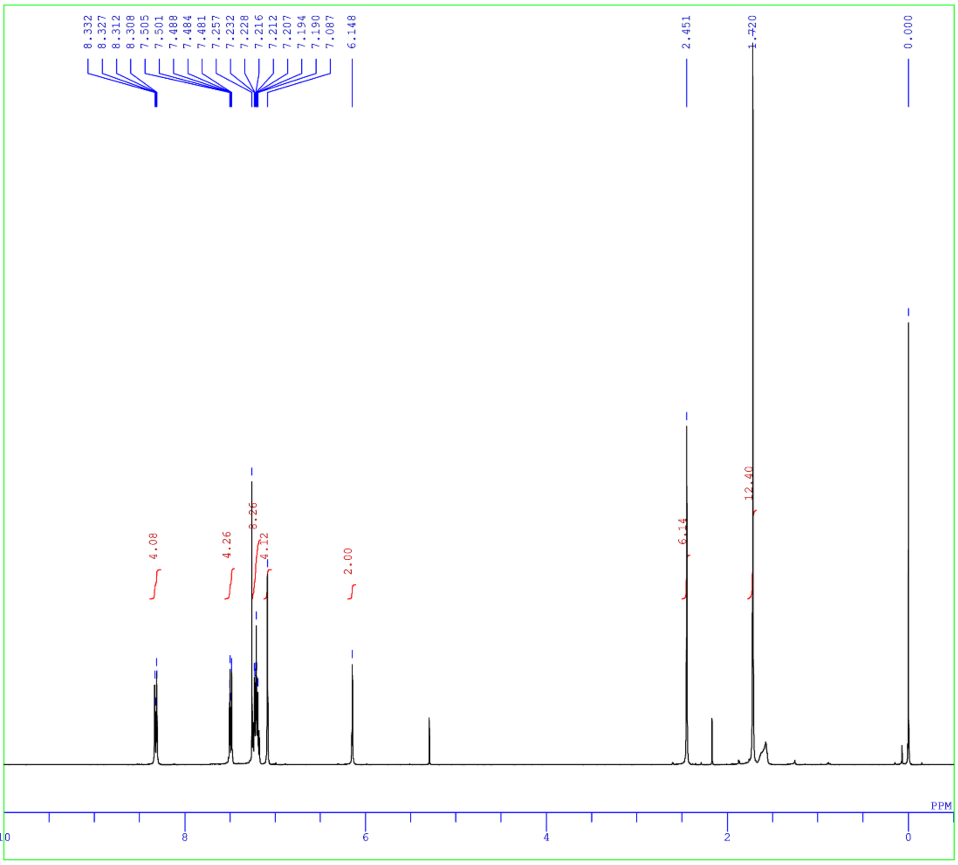

^{13}C NMR (100 MHz) of compound **4** ($CDCl_3$)

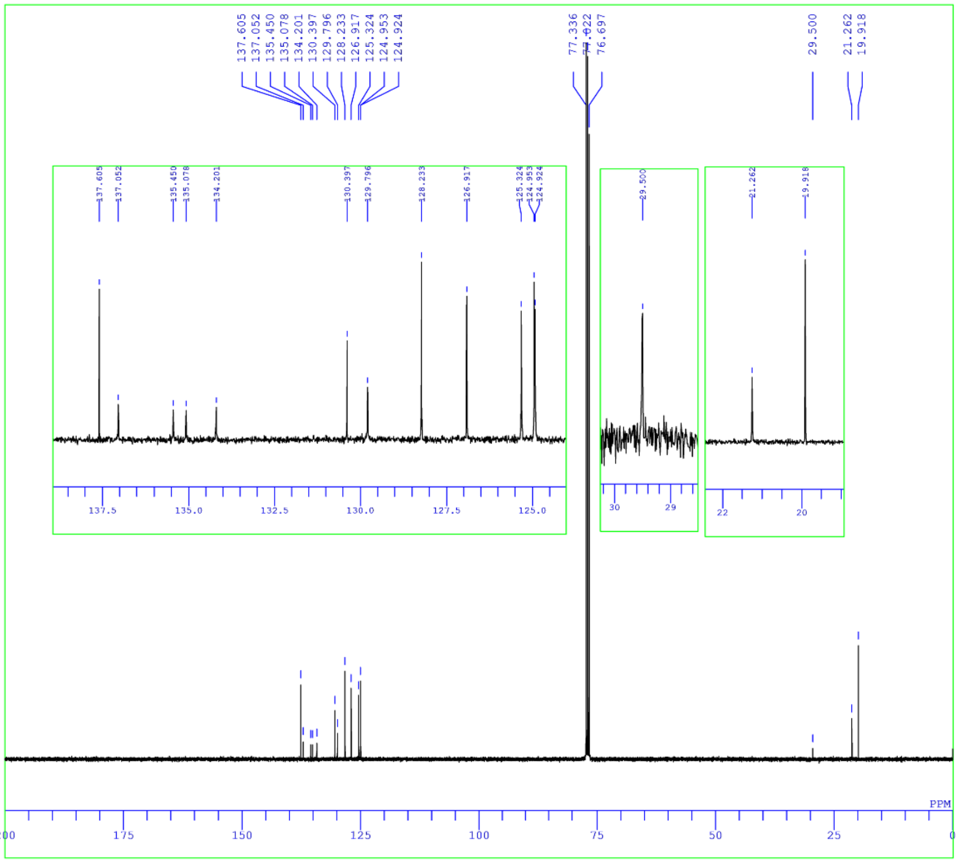

^{1}H NMR (400 MHz) of compound DAntM cation (TFA-*d*)

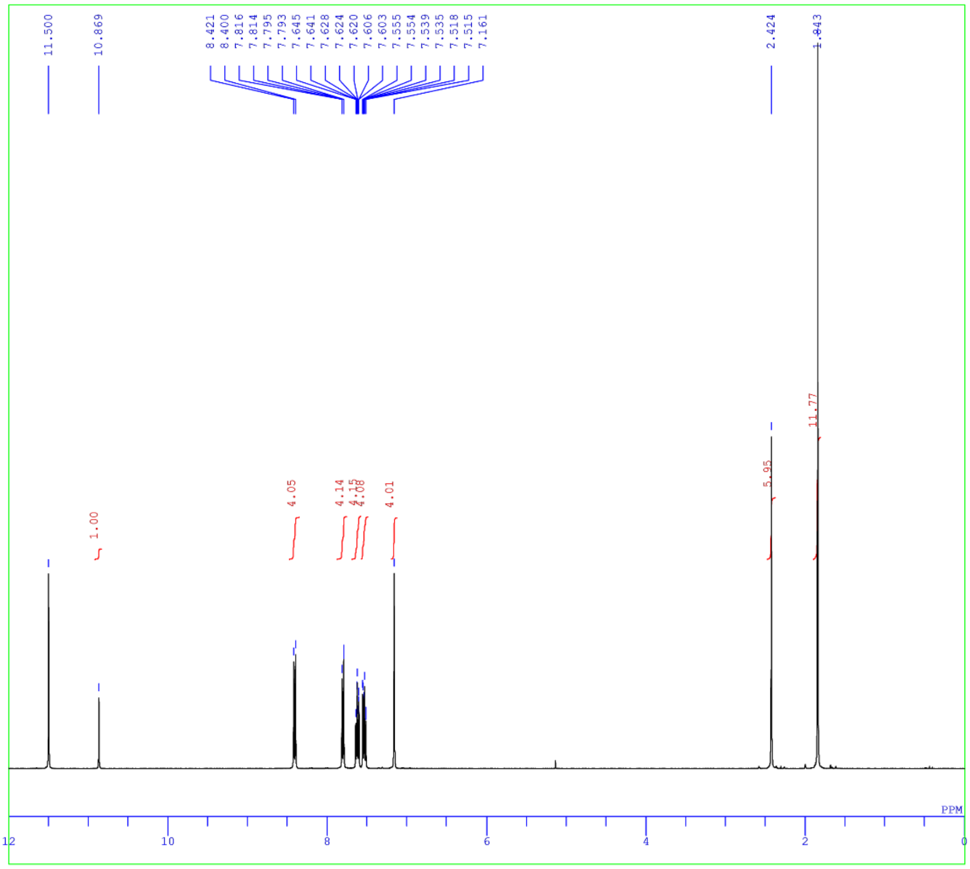

^{13}C NMR (100 MHz) of compound DAntM cation (TFA-*d*)

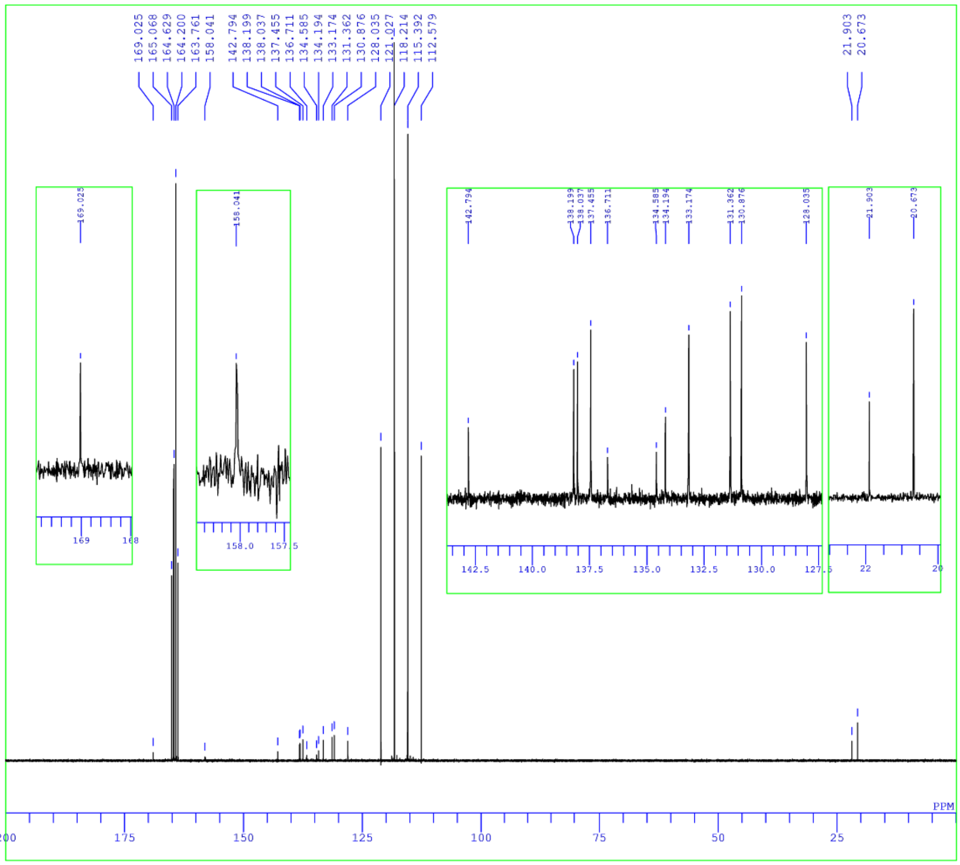

Cartesian coordinates of optimized structures

Table S2. Cartesian coordinates of the DAntM radical (spin localization at the central sp^2 carbon)

Center Number	Atomic Number	Atomic Type	Coordinates (Angstroms) X	Y	Z
1	6	0	-3.261607	3.618518	1.930141
2	6	0	-4.582034	3.125241	1.806423
3	6	0	-4.809636	1.952928	1.129991
4	6	0	-3.741933	1.225600	0.518535
5	6	0	-2.404558	1.751760	0.596192
6	6	0	-2.210436	2.940417	1.355747
7	6	0	-3.987840	-0.011125	-0.123383
8	6	0	-2.926607	-0.707578	-0.748863
9	6	0	-1.591137	-0.168646	-0.743350
10	6	0	-1.303777	1.051050	-0.024447
11	6	0	-3.172494	-1.941749	-1.427857
12	6	0	-2.176434	-2.592630	-2.110834
13	6	0	-0.881727	-2.024703	-2.171558
14	6	0	-0.606536	-0.849056	-1.512627
15	6	0	-0.003473	1.633007	0.032446
16	6	0	1.304661	1.044397	0.059714
17	6	0	1.603552	-0.165453	0.780990
18	6	0	2.942277	-0.698969	0.773883
19	6	0	3.990083	-0.005548	0.125288
20	6	0	3.728273	1.220244	-0.528096
21	6	0	2.386420	1.739604	-0.586846
22	6	0	4.780209	1.946518	-1.168974
23	6	0	4.532304	3.109228	-1.853433
24	6	0	3.206124	3.596025	-1.956764
25	6	0	2.170152	2.920819	-1.355013
26	6	0	0.627512	-0.848791	1.561527
27	6	0	0.915996	-2.017835	2.223772
28	6	0	2.215412	-2.577730	2.156640
29	6	0	3.201359	-1.926941	1.460617
30	6	0	-5.370007	-0.581871	-0.139979
31	6	0	5.375156	-0.570000	0.126986
32	6	0	-6.243684	-0.270714	-1.201042
33	6	0	-7.533223	-0.813871	-1.197218
34	6	0	-7.951163	-1.650641	-0.164223
35	6	0	-7.080568	-1.954465	0.880676
36	6	0	-5.784823	-1.427331	0.908511
37	6	0	6.264525	-0.240647	1.169287
38	6	0	7.556411	-0.777959	1.151537
39	6	0	7.961378	-1.626611	0.123091
40	6	0	7.075249	-1.948419	-0.903200
41	6	0	5.776717	-1.427605	-0.916791
42	6	0	4.814066	-1.774661	-2.027817
43	6	0	5.821946	0.677383	2.283843
44	6	0	-5.786903	0.634488	-2.320343
45	6	0	-4.838921	-1.754620	2.039711
46	1	0	-3.073107	4.526502	2.495291
47	1	0	-5.408381	3.662271	2.262448
48	1	0	-5.814109	1.551732	1.052653
49	1	0	-1.205815	3.314834	1.510906
50	1	0	-4.176910	-2.349553	-1.403532
51	1	0	-2.385521	-3.527931	-2.621614
52	1	0	-0.101675	-2.512855	-2.748122
53	1	0	0.385119	-0.426152	-1.590668
54	1	0	0.001273	2.717411	0.063835
55	1	0	5.788312	1.551726	-1.106862
56	1	0	5.346380	3.645456	-2.331952
57	1	0	3.003530	4.496901	-2.528410
58	1	0	1.159401	3.286382	-1.491953
59	1	0	-0.366429	-0.431330	1.642649
60	1	0	0.144180	-2.509040	2.808806
61	1	0	2.434056	-3.508126	2.672352
62	1	0	4.207556	-2.329909	1.429210
63	1	0	-8.211887	-0.577235	-2.012424
64	1	0	-8.954840	-2.066117	-0.173647
65	1	0	-7.406177	-2.607528	1.686064
66	1	0	8.247182	-0.527357	1.952271
67	1	0	8.967041	-2.037362	0.121575
68	1	0	7.390747	-2.610771	-1.705015
69	1	0	5.271708	-2.453767	-2.751873
70	1	0	3.911336	-2.252600	-1.631831
71	1	0	4.486808	-0.875126	-2.560565
72	1	0	5.513489	1.652892	1.892490
73	1	0	4.957679	0.263454	2.814516
74	1	0	6.624418	0.837450	3.008602
75	1	0	-4.913031	0.216856	-2.832065
76	1	0	-6.578464	0.782289	-3.059562
77	1	0	-5.488001	1.616077	-1.936843
78	1	0	-5.304844	-2.426798	2.764952
79	1	0	-3.927187	-2.232618	1.664991
80	1	0	-4.525406	-0.846406	2.565982

Table S3. Cartesian coordinates of the DAntM radical (localization on the anthryl group)

Center Number	Atomic Number	Atomic Type	Coordinates (Angstroms) X	Y	Z
1	6	0	-3.251717	3.946950	1.154492
2	6	0	-4.520867	3.307601	1.222442
3	6	0	-4.675608	2.028879	0.756205
4	6	0	-3.580320	1.311170	0.176665
5	6	0	-2.298654	1.968238	0.084043
6	6	0	-2.177084	3.292959	0.611423
7	6	0	-3.723622	-0.017622	-0.271312
8	6	0	-2.630593	-0.684790	-0.862267
9	6	0	-1.353736	-0.022092	-0.985005
10	6	0	-1.188884	1.287311	-0.467126
11	6	0	-2.755992	-2.027673	-1.346189
12	6	0	-1.701548	-2.673828	-1.933174
13	6	0	-0.452943	-2.009754	-2.086069
14	6	0	-0.288789	-0.729434	-1.629694
15	6	0	0.127805	1.956303	-0.521960
16	6	0	1.294288	1.529914	0.056237
17	6	0	1.369517	0.369551	0.954878
18	6	0	2.557124	-0.426406	0.954016
19	6	0	3.709469	0.002218	0.225406
20	6	0	3.752832	1.322977	-0.321039
21	6	0	2.576331	2.138982	-0.332415
22	6	0	4.956903	1.862876	-0.850726
23	6	0	5.018465	3.164560	-1.309284
24	6	0	3.875245	3.981158	-1.260179
25	6	0	2.676890	3.465490	-0.785694
26	6	0	0.305632	-0.002243	1.795589
27	6	0	0.358515	-1.160647	2.557691
28	6	0	1.491544	-1.990428	2.495840
29	6	0	2.571990	-1.627010	1.715360
30	6	0	-5.025663	-0.731517	-0.089864
31	6	0	4.876551	-0.914980	0.078933
32	6	0	-6.013624	-0.664904	-1.091664
33	6	0	-7.221789	-1.343665	-0.896569
34	6	0	-7.447621	-2.075946	0.267329
35	6	0	-6.464289	-2.137624	1.253088
36	6	0	-5.245606	-1.469899	1.090533
37	6	0	5.940578	-0.874672	1.002214
38	6	0	7.015826	-1.754447	0.833533
39	6	0	7.036441	-2.660002	-0.225456
40	6	0	5.976477	-2.698924	-1.129707
41	6	0	4.886489	-1.833325	-0.991037
42	6	0	3.731571	-1.876505	-1.965071
43	6	0	5.907651	0.099510	2.156071
44	6	0	-5.763604	0.127658	-2.352733
45	6	0	-4.178703	-1.533253	2.158824
46	1	0	-3.135501	4.954221	1.543590
47	1	0	-5.365800	3.834546	1.655877
48	1	0	-5.638468	1.533641	0.820794
49	1	0	-1.205427	3.775119	0.584741
50	1	0	-3.714175	-2.524447	-1.240196
51	1	0	-1.818879	-3.691626	-2.293498
52	1	0	0.373617	-2.519509	-2.572643
53	1	0	0.664300	-0.232201	-1.758066
54	1	0	0.173730	2.836306	-1.159795
55	1	0	5.840651	1.234317	-0.872578
56	1	0	5.953673	3.559693	-1.694694
57	1	0	3.926888	5.014075	-1.590731
58	1	0	1.802703	4.107593	-0.745375
59	1	0	-0.571650	0.629907	1.849247
60	1	0	-0.477607	-1.422088	3.199217
61	1	0	1.527856	-2.906916	3.077453
62	1	0	3.462049	-2.246372	1.690598
63	1	0	-7.988391	-1.296334	-1.665503
64	1	0	-8.389607	-2.598946	0.406095
65	1	0	-6.640695	-2.709267	2.160445
66	1	0	7.839760	-1.728575	1.541802
67	1	0	7.877176	-3.337540	-0.344481
68	1	0	5.991472	-3.407958	-1.953270
69	1	0	3.893783	-2.630783	-2.739645
70	1	0	2.790627	-2.107070	-1.452491
71	1	0	3.593177	-0.906667	-2.455875
72	1	0	5.879521	1.134933	1.799659
73	1	0	5.011250	-0.047622	2.768254
74	1	0	6.784158	-0.016539	2.798828
75	1	0	-4.882658	-0.247238	-2.884959
76	1	0	-6.620264	0.077407	-3.029863
77	1	0	-5.568160	1.180996	-2.124188
78	1	0	-4.496214	-2.156124	2.999191
79	1	0	-3.243399	-1.942024	1.760933
80	1	0	-3.946747	-0.533211	2.541664

Table S4. Cartesian coordinates of the DAntM dimer

Center	Atomic	Atomic	Coordinates (Angstroms)		
Number	Number	Type	X	Y	Z
1	6	0	3.149975	-0.659105	3.223149
2	6	0	4.384364	-1.329794	3.025462
3	6	0	4.598759	-2.007161	1.857355
4	6	0	3.601812	-2.071719	0.829943
5	6	0	2.332502	-1.401603	1.019789
6	6	0	2.176342	-0.694471	2.261047
7	6	0	3.858139	-2.787213	-0.353721
8	6	0	2.866000	-2.868394	-1.344644
9	6	0	1.577987	-2.235351	-1.148064
10	6	0	1.313306	-1.483608	0.023576
11	6	0	3.124985	-3.573227	-2.565297
12	6	0	2.186409	-3.662458	-3.555461
13	6	0	0.913943	-3.060837	-3.365557
14	6	0	0.627552	-2.387873	-2.208219
15	6	0	-0.024321	-0.742208	0.287531
16	6	0	-1.327334	-1.583515	0.112182
17	6	0	-1.585433	-2.531192	1.147131
18	6	0	-2.854806	-3.217826	1.230379
19	6	0	-3.850592	-2.981651	0.267280
20	6	0	-3.589357	-2.106323	-0.799857
21	6	0	-2.320643	-1.406330	-0.893957
22	6	0	-4.592670	-1.905940	-1.805150
23	6	0	-4.385351	-1.089314	-2.880205
24	6	0	-3.140349	-0.422699	-3.007646
25	6	0	-2.165612	-0.578392	-2.059902
26	6	0	-0.634160	-2.834135	2.178944
27	6	0	-0.908749	-3.705957	3.200279
28	6	0	-2.165528	-4.359317	3.278934
29	6	0	-3.104762	-4.120210	2.314322
30	6	0	3.150000	0.659048	-3.223115
31	6	0	4.384396	1.329722	-3.025427
32	6	0	4.598802	2.007081	-1.857317
33	6	0	3.601875	2.071598	-0.829882
34	6	0	2.332567	1.401475	-1.019720
35	6	0	2.176375	0.694403	-2.261004
36	6	0	3.858209	2.787085	0.353785
37	6	0	2.866095	2.868206	1.344740
38	6	0	1.578088	2.235143	1.148175
39	6	0	1.313387	1.483444	-0.023488
40	6	0	3.125097	3.573013	2.565405
41	6	0	2.186540	3.662207	3.555591
42	6	0	0.914079	3.060575	3.365698
43	6	0	0.627672	2.387632	2.208352
44	6	0	-0.024272	0.742108	-0.287486
45	6	0	-1.327246	1.583478	-0.112172
46	6	0	-1.585280	2.531183	-1.147110
47	6	0	-2.854624	3.217869	-1.230375
48	6	0	-3.850442	2.981719	-0.267302
49	6	0	-3.589265	2.106365	0.799829
50	6	0	-2.320581	1.406322	0.893943
51	6	0	-4.592605	1.906003	1.805099
52	6	0	-4.385336	1.089349	2.880143
53	6	0	-3.140360	0.422687	3.007600
54	6	0	-2.165598	0.578362	2.059877
55	6	0	-0.633966	2.834111	-2.178892
56	6	0	-0.908499	3.705955	-3.200222
57	6	0	-2.165257	4.359355	-3.278904
58	6	0	-3.104522	4.120272	-2.314316
59	6	0	5.177117	3.468815	0.547676
60	6	0	-5.178226	3.665581	-0.372624
61	6	0	-5.178410	-3.665451	0.372585
62	6	0	5.177092	-3.468831	-0.547678
63	6	0	6.228360	2.786579	1.191820
64	6	0	7.456158	3.437521	1.356137
65	6	0	7.640732	4.739113	0.893994
66	6	0	6.594497	5.407540	0.260856
67	6	0	5.353994	4.786147	0.079255
68	6	0	-5.354930	4.940658	0.200608
69	6	0	-6.603926	5.562643	0.094086
70	6	0	-7.658742	4.935211	-0.566051
71	6	0	-7.475298	3.673615	-1.128856
72	6	0	-6.239493	3.023214	-1.041268
73	6	0	5.354209	-4.786046	-0.079017
74	6	0	6.594756	-5.407325	-0.260703
75	6	0	7.640800	-4.738904	-0.894162
76	6	0	7.455988	-3.437431	-1.356544
77	6	0	6.228141	-2.786601	-1.192145
78	6	0	-6.239666	-3.023014	1.041179
79	6	0	-7.475508	-3.673349	1.128743
80	6	0	-7.658996	-4.934951	0.565965

Center Number	Atomic Number	Atomic Type	X	Y	Z
81	6	0	-6.604191	-5.562455	-0.094119
82	6	0	-5.355160	-4.940536	-0.200617
83	6	0	4.219984	5.507445	-0.609905
84	6	0	6.018082	1.383952	1.704613
85	6	0	-4.211397	-5.617724	-0.917829
86	6	0	-6.036235	-1.656149	1.646541
87	6	0	6.017591	-1.384132	-1.705264
88	6	0	4.220411	-5.507335	0.610503
89	6	0	-6.036107	1.656362	-1.646672
90	6	0	-4.211156	5.617771	0.917873
91	1	0	2.973415	-0.099684	4.136819
92	1	0	5.153514	-1.295340	3.790904
93	1	0	5.539669	-2.517226	1.685445
94	1	0	1.270330	-0.145041	2.465530
95	1	0	4.097776	-4.035852	-2.688774
96	1	0	2.406589	-4.195578	-4.475527
97	1	0	0.154878	-3.139034	-4.138563
98	1	0	-0.352854	-1.966026	-2.099862
99	1	0	0.000137	-0.598612	1.356590
100	1	0	-5.531568	-2.436146	-1.695453
101	1	0	-5.158745	-0.955667	-3.630112
102	1	0	-2.949956	0.221269	-3.860941
103	1	0	-1.236640	-0.067794	-2.239314
104	1	0	0.349854	-2.396930	2.155085
105	1	0	-0.148111	-3.902856	3.950296
106	1	0	-2.374883	-5.046222	4.093454
107	1	0	-4.069662	-4.613025	2.353926
108	1	0	2.973422	0.099655	-4.136798
109	1	0	5.153532	1.295290	-3.790885
110	1	0	5.539699	2.517174	-1.685421
111	1	0	1.270346	0.145006	-2.465501
112	1	0	4.097889	4.035635	2.688879
113	1	0	2.406732	4.195304	4.475667
114	1	0	0.155025	3.138752	4.138718
115	1	0	-0.352732	1.965775	2.100008
116	1	0	0.000215	0.598521	-1.356547
117	1	0	-5.531482	2.436244	1.695391
118	1	0	-5.158750	0.955718	3.630033
119	1	0	-2.950007	-0.221301	3.860888
120	1	0	-1.236645	0.067729	2.239295
121	1	0	0.350035	2.396877	-2.155005
122	1	0	-0.147836	3.902841	-3.950216
123	1	0	-2.374569	5.046272	-4.093423
124	1	0	-4.069404	4.613122	-2.353938
125	1	0	8.271150	2.916165	1.851313
126	1	0	8.599146	5.232857	1.027671
127	1	0	6.737612	6.423270	-0.098303
128	1	0	-6.747066	6.545922	0.534359
129	1	0	-8.623408	5.429115	-0.641272
130	1	0	-8.297828	3.183637	-1.643235
131	1	0	6.738055	-6.422963	0.098644
132	1	0	8.599251	-5.232559	-1.027902
133	1	0	8.270832	-2.916078	-1.851967
134	1	0	-8.298030	-3.183315	1.643080
135	1	0	-8.623691	-5.428803	0.641166
136	1	0	-6.747365	-6.545739	-0.534368
137	1	0	4.509130	6.523057	-0.892351
138	1	0	3.907394	4.975611	-1.515241
139	1	0	3.339064	5.568247	0.038026
140	1	0	5.679421	0.710060	0.912082
141	1	0	6.935117	0.971531	2.133333
142	1	0	5.238103	1.355793	2.472077
143	1	0	-4.515082	-6.586535	-1.322832
144	1	0	-3.843302	-5.000014	-1.744140
145	1	0	-3.362892	-5.778468	-0.244002
146	1	0	-6.940561	-1.302409	2.148624
147	1	0	-5.217892	-1.661308	2.373127
148	1	0	-5.757465	-0.917880	0.887486
149	1	0	6.934704	-0.971406	-2.133523
150	1	0	5.238051	-1.356423	-2.473201
151	1	0	5.678167	-0.710253	-0.913055
152	1	0	4.509655	-6.522935	0.892895
153	1	0	3.908088	-4.975479	1.515919
154	1	0	3.339298	-5.568165	-0.037161
155	1	0	-5.757279	0.918078	-0.887654
156	1	0	-6.940468	1.302633	-2.148698
157	1	0	-5.217816	1.661538	-2.373318
158	1	0	-4.514809	6.586579	1.322905
159	1	0	-3.843105	5.000015	1.744169
160	1	0	-3.362629	5.778505	0.244071

Table S5. Cartesian coordinates of the DAntM cation

Center Number	Atomic Number	Atomic Type	Coordinates (Angstroms) X	Y	Z
1	6	0	-3.121460	3.596904	1.898735
2	6	0	-4.466945	3.182946	1.782525
3	6	0	-4.758344	2.012777	1.125746
4	6	0	-3.727000	1.224562	0.532117
5	6	0	-2.361878	1.667675	0.607401
6	6	0	-2.100453	2.848776	1.348678
7	6	0	-4.042399	-0.006466	-0.091524
8	6	0	-3.025278	-0.789257	-0.690346
9	6	0	-1.655396	-0.346757	-0.679696
10	6	0	-1.305422	0.881237	0.003146
11	6	0	-3.365429	-2.011064	-1.346309
12	6	0	-2.414003	-2.756377	-1.997269
13	6	0	-1.082439	-2.288410	-2.044758
14	6	0	-0.718853	-1.118067	-1.411367
15	6	0	-0.000000	1.433156	0.000091
16	6	0	1.305419	0.881208	-0.003029
17	6	0	1.655416	-0.346769	0.679827
18	6	0	3.025315	-0.789219	0.690499
19	6	0	4.042401	-0.006474	0.091557
20	6	0	3.726960	1.224496	-0.532175
21	6	0	2.361842	1.667627	-0.607356
22	6	0	4.758254	2.012649	-1.125983
23	6	0	4.466799	3.182778	-1.782802
24	6	0	3.121308	3.596774	-1.898869
25	6	0	2.100351	2.848703	-1.348653
26	6	0	0.718887	-1.118099	1.411492
27	6	0	1.082522	-2.288382	2.044969
28	6	0	2.414118	-2.756260	1.997577
29	6	0	3.365519	-2.010947	1.346582
30	6	0	-5.457860	-0.483014	-0.115000
31	6	0	5.457870	-0.483007	0.114979
32	6	0	-6.292071	-0.118792	-1.191281
33	6	0	-7.614487	-0.574602	-1.190879
34	6	0	-8.096153	-1.369573	-0.152800
35	6	0	-7.259224	-1.721783	0.904213
36	6	0	-5.930681	-1.286441	0.942214
37	6	0	6.292193	-0.118595	1.191105
38	6	0	7.614623	-0.574367	1.190624
39	6	0	8.096193	-1.369478	0.152608
40	6	0	7.259153	-1.721875	-0.904254
41	6	0	5.930591	-1.286584	-0.942167
42	6	0	5.021641	-1.666431	-2.087602
43	6	0	5.767969	0.742268	2.316334
44	6	0	-5.767749	0.741911	-2.316585
45	6	0	-5.021826	-1.666145	2.087771
46	1	0	-2.883255	4.505011	2.443785
47	1	0	-5.259150	3.775498	2.227392
48	1	0	-5.780839	1.661336	1.053692
49	1	0	-1.079965	3.168893	1.520824
50	1	0	-4.400886	-2.329930	-1.333253
51	1	0	-2.685132	-3.682807	-2.492213
52	1	0	-0.333546	-2.850413	-2.594175
53	1	0	0.304852	-0.782620	-1.490632
54	1	0	0.000015	2.518492	0.000121
55	1	0	5.780749	1.661189	-1.054025
56	1	0	5.258959	3.775282	-2.227816
57	1	0	2.883075	4.504858	-2.443947
58	1	0	1.079842	3.168830	-1.520669
59	1	0	-0.304844	-0.782718	1.490698
60	1	0	0.333638	-2.850408	2.594377
61	1	0	2.685286	-3.682628	2.492616
62	1	0	4.400995	-2.329757	1.333572
63	1	0	-8.268726	-0.301620	-2.013683
64	1	0	-9.125139	-1.715104	-0.167534
65	1	0	-7.637037	-2.341379	1.712273
66	1	0	8.268945	-0.301245	2.013316
67	1	0	9.125192	-1.714977	0.167277
68	1	0	7.636893	-2.341587	-1.712260
69	1	0	5.543365	-2.292611	-2.814379
70	1	0	4.144703	-2.220162	-1.733375
71	1	0	4.650009	-0.779214	-2.612615
72	1	0	5.414364	1.711196	1.946408
73	1	0	4.921230	0.264867	2.822173
74	1	0	6.542428	0.930324	3.062836
75	1	0	-4.921082	0.264356	-2.822401
76	1	0	-6.542193	0.930008	-3.063093
77	1	0	-5.414009	1.710822	-1.946745
78	1	0	-5.543704	-2.291952	2.814758
79	1	0	-4.145045	-2.220233	1.733706
80	1	0	-4.649941	-0.778864	2.612490

Table S6. Cartesian coordinates of anthroxyl radical **5**

Center Number	Atomic Number	Atomic Type	Coordinates (Angstroms) X	Y	Z
1	6	0	3.683603	-0.000000	1.938640
2	6	0	3.681188	-0.000000	0.532527
3	6	0	2.487114	-0.000000	-0.167507
4	6	0	1.241656	-0.000000	0.515662
5	6	0	1.259080	-0.000000	1.942388
6	6	0	2.478677	-0.000000	2.630155
7	6	0	0.000000	0.000000	-0.197189
8	6	0	-1.241656	0.000000	0.515662
9	6	0	-1.259080	0.000000	1.942388
10	6	0	0.000000	-0.000000	2.717188
11	6	0	-2.487114	0.000000	-0.167507
12	6	0	-3.681188	0.000000	0.532527
13	6	0	-3.683603	0.000000	1.938640
14	6	0	-2.478677	0.000000	2.630155
15	8	0	0.000000	-0.000000	3.954199
16	6	0	-0.000000	0.000000	-1.689778
17	6	0	-0.000000	-1.225677	-2.385819
18	6	0	-0.000000	-1.206811	-3.784756
19	6	0	-0.000000	0.000000	-4.481523
20	6	0	-0.000000	1.206811	-3.784756
21	6	0	0.000000	1.225677	-2.385819
22	6	0	-0.000000	-2.531589	-1.626997
23	6	0	0.000000	2.531589	-1.626997
24	1	0	4.624164	-0.000000	2.480976
25	1	0	4.622026	-0.000000	-0.010088
26	1	0	2.489336	-0.000000	-1.252137
27	1	0	2.445141	-0.000000	3.714534
28	1	0	-2.489336	0.000000	-1.252137
29	1	0	-4.622026	0.000000	-0.010088
30	1	0	-4.624164	0.000000	2.480976
31	1	0	-2.445141	0.000000	3.714534
32	1	0	-0.000000	-2.147764	-4.328363
33	1	0	-0.000000	0.000000	-5.567724
34	1	0	0.000000	2.147764	-4.328363
35	1	0	-0.879104	-2.611480	-0.978175
36	1	0	-0.000000	-3.386358	-2.307976
37	1	0	0.879104	-2.611480	-0.978175
38	1	0	0.000000	3.386358	-2.307976
39	1	0	-0.879104	2.611480	-0.978175
40	1	0	0.879104	2.611480	-0.978175

References

[S1] Zhen, C. –J.; Lu, S. –F.; Lin, M. –H.; Wu, J. –T.; Chao, I.; Lin, C. –H. *Chem. Eur. J.* **2021**, 27, 16682-16689.

[S2] Lü, J. –M.; Rosokha, S. V.; Kochi, J. K. *J. Am. Chem. Soc*. **2003**, *125*, 12161-12171.
